# Microbial dysbiosis in roots and rhizosphere of grapevines experiencing decline is associated with active metabolic functions

**DOI:** 10.3389/fpls.2024.1358213

**Published:** 2024-04-02

**Authors:** Romain Darriaut, Tania Marzari, Vincent Lailheugue, Joseph Tran, Guilherme Martins, Elisa Marguerit, Isabelle Masneuf-Pomarède, Virginie Lauvergeat

**Affiliations:** ^1^ EGFV, Univ. Bordeaux, Bordeaux Sciences Agro, INRAE, ISVV, Villenave d’Ornon, France; ^2^ Université de Bordeaux, INRAE, Bordeaux INP, Bordeaux Sciences Agro, UMR Œnologie 1366, ISVV, Villenave d’Ornon, France; ^3^ Bordeaux Sciences Agro, 1 cours du Général de Gaulle, Gradignan, France

**Keywords:** grapevine fitness, soil quality, metabarcoding-based predicted functionality, root endophytes, belowground microbiome, *Vitis vinifera* growth

## Abstract

When grapevine decline, characterized by a premature decrease in vigor and yield and sometimes plant death, cannot be explained by pathological or physiological diseases, one may inquire whether the microbiological status of the soil is responsible. Previous studies have shown that the composition and structure of bacterial and fungal microbial communities in inter-row soil are affected in areas displaying vine decline, compared to areas with non-declining vines within the same plot. A more comprehensive analysis was conducted in one such plot. Although soil chemical parameters could not directly explain these differences, the declining vines presented lower vigor, yield, berry quality, and petiole mineral content than those in non-declining vines. The bacterial and fungal microbiome of the root endosphere, rhizosphere, and different horizons of the bulk soil were explored through enzymatic, metabolic diversity, and metabarcoding analysis in both areas. Despite the lower microbial diversity and richness in symptomatic roots and soil, higher microbial activity and enrichment of potentially both beneficial bacteria and pathogenic fungi were found in the declining area. Path modeling analysis linked the root microbial activity to berry quality, suggesting a determinant role of root microbiome in the berry mineral content. Furthermore, certain fungal and bacterial taxa were correlated with predicted metabolic pathways and metabolic processes assessed with Eco-Plates. These results unexpectedly revealed active microbial profiles in the belowground compartments associated with stressed vines, highlighting the interest of exploring the functional microbiota of plants, and more specifically roots and rhizosphere, under stressed conditions.

## 1 Introduction

Plant decline is defined as the decrease in physiological processes of the plant, leading to the loss of vigor and sometimes death. In perennial crops, this decline corresponds to an economic loss related to productivity downsizing and is mainly linked to pathogenic causes (Cox et al., 2005; Chuche et al., 2018). Pathogen predisposition to invade crops is facilitated by climate change phenomenon and undoubtedly by the loss of soil resilience (Chakraborty et al., 2008; Cienciala et al., 2017). Temperature variations, salinity level, and water deficiency disturb the ecosystem processes and lead to optimal growth conditions for opportunistic and sometimes pathogenic microorganisms (Marçais and Bréda, 2006; Fraser and Brown, 2017; Pandey and Senthil-Kumar, 2019).

Fungi and bacteria are the most dominant telluric microorganisms, and numerous descriptive analyses have been conducted in diverse habitats and on a variety of crops (Karimi et al., 2020). Soil microorganisms trigger all ecosystem processes, including nutrient cycling, litter decomposition, soil remediation, and phytopathogen biocontrol (Fierer, 2017). Optimal soil biogeochemical services are therefore dependent on a state of equilibrium among keystone taxa (Banerjee et al., 2018). Agricultural practices, in addition to pedoclimatic conditions, are one of the most important drivers for microbial communities and can easily lead to a dysregulation of those services and result in decline features (Banerjee et al., 2019; Lin et al., 2019). This decline can be associated with microbial dysbiosis within the host, wherein the host’s ability to regulate its microbiome is compromised, consequently diminishing host fitness by undermining functional stability (Arnault et al., 2023). Microbial dysbiosis has already been proven to be linked to the fitness of various crops such as tomato (Lee et al., 2021), banana (Köberl et al., 2017), and citrus (Ginnan et al., 2020). For instance, the incidence of grapevine trunk diseases (GTDs), which are the most studied fungal pathogens related to grapevine decline, are mainly influenced by soil management practices and edaphic parameters (Gramaje et al., 2018). On a global level, vineyards are believed to contain some of the poorest soils in terms of biodiversity across agricultural systems due to the intensive use of chemical treatments (Karimi et al., 2019).

Soil microbiome shapes the plant-associated microbiome and has a strong influence on its fitness (Wei et al., 2019). There is evidence showing the impacts of taxonomic and functional diversity on plant health and productivity (Chen et al., 2020; Trivedi et al., 2020). The composition of fungal and/or bacterial communities has been extensively investigated in vineyards through the soil, rhizosphere, and/or root compartments under various spatial, temporal, and rootstock-genotype dynamics (ZarraonaIndia et al., 2015; D’Amico et al., 2018; Marasco et al., 2022; Nanetti et al., 2023). The assembly of endophytic communities was also explored under different types of pedoclimatic conditions (Deyett and Rolshausen, 2019; Pacifico et al., 2019; Bekris et al., 2021; Liu and Howell, 2021). Much research has been performed on GTD issues (Cobos et al., 2022), highlighting the evidence that soil acts as an important source of pathogen inoculum for grapevines (Nerva et al., 2019). However, besides GTDs, few authors have analyzed the relationship between grapevine decline and the plant-associated microbiome in dysbiosis (Bettenfeld et al., 2022). Different authors have shed light on rhizosphere and root microbiome of grapevine subjected to drought (Carbone et al., 2021) and salt stress (Wang et al., 2023). However, no research has been conducted on the microbiome in the case of grapevine decline with symptoms unrelated to pathogen infection or known abiotic stress.

A first study was conducted at four French vineyards showing vine decline symptoms in areas located in the middle of a plot [i.e., low vigor and high mortality, described as symptomatic (S) areas], in which no clear mineral deficiency or specific disease was detected (Darriaut et al., 2021). Among the studied plots, bulk soils in the S areas showed different bacterial and fungal profiles with increased metabolic activities (Darriaut et al., 2021) combined with higher abundances of putative pathogenic fungi and potentially beneficial bacteria compared to asymptomatic (AS) areas (Darriaut et al., 2023). The effect of the microbial composition of both soils harvested from one of these plots on young grapevine plant growth and rhizosphere and roots was investigated in a greenhouse experiment (Darriaut et al., 2022a), confirming the microbial dysbiosis inherent to the observed decline. However, the microbiome associated with grapevine roots *in situ* (i.e., within the plot) has not been analyzed to date, and one can wonder if the microbial dysregulation would occur in this belowground compartment.

The aim of the present study was to investigate the influence of the soil microbiome on the rhizosphere and root microbial composition, as well as plant responses, in a deeper and holistic analysis of soil and vine plants in both S and AS areas located within a same vineyard plot. The working hypothesis put forward is that root microbial communities, like those in the rhizosphere, are subject to both bacterial and fungal imbalance.

This work was carried out on a plot in which dysbiosis was detected in the inter-row soil taken from the declining zone in 2018 and 2019 (Darriaut et al., 2021: Darriaut et al., 2023). For the present study, new samples were collected in 2020 and 2021, as well as more in-depth phenotypic analyses. Microbiological analyses were performed in the different soil horizons, as well as in the rhizosphere and the root endosphere of the grapevines in the two areas. Microbial communities were studied using a cultivable approach and amplicon-based sequencing for fungal ITS and the bacterial 16S rRNA gene.

## 2 Materials and methods

### 2.1 Studied site

The studied vineyard located in the Graves appellation near Bordeaux (France) was depicted in Darriaut et al. (2021) as plot number 2. *Vitis vinifera* Cabernet-Sauvignon scion grafted on *V. riparia* Gloire de Montpellier rootstock (CS/RGM) vines was planted in sandy soil in 2010. The vineyard is located in a sub-humid temperate climate subtyped as Cfb in Köppen climate classification and characterized by cool nights and few extreme temperatures. Delimited areas displaying grapevine decline, referred to as the symptomatic area (S), and the healthy, asymptomatic area (AS), located 10 m apart from each other (around 60 m² each, with 4 rows having 15 vines per row), were identified in the same plot (**Supplementary Figure S1**). Both S and AS areas were cover cropped with oats and crimson clover and treated with organic amendments. The amendments used were 0.8 t/ha of Bochevo® [3% total nitrogen (N), 2.5% N_organic_, 2.5% phosphate (P_2_O_5_), 2.8% potassium oxide (K_2_O), 6.5% lime (CaO), and 1% magnesia (MgO)] and Touban® (0.9% total nitrogen, 0.88% N_organic_, 0.6% K_2_O, 0.2% P_2_O_5_, 0.2% MgO, and 2.3% CaO, pH ~ 7.5). The S area was characterized by high mortality rate (57% dead or vines recently planted to replace dead ones in the S area compared to 1% in the AS area) and low vigor of the established vines (2.2 times less pruning weight per vine in the S area compared to the AS area, *p* < 0.01), which were not linked to disease symptoms related to the main viruses (GFLV or ArMV) or mineral deficiencies.

### 2.2 Plant phenotyping, must quality evaluation, vine water status, and mineral content of the leaves

In both areas, the oldest vines (approximately 10 years old) were phenotyped and sampled in 2020. Primary and secondary bunches of grapes from 28 vines spread over four rows for both AS and S areas were collected and weighed. The average bunch weight was calculated as the weight of the bunches divided by the number of bunches for each vine.

Approximately 10 berries of the same size were collected per vine from all over the bunches for a total of 100 berries in triplicate for each of the S and AS areas. The sampling was done in bags with a vertical filter (BagFilter® 400 mL, Interscience). These 100 berries were weighed, and the average berry mass was calculated. The juice was extracted from the berries using a crusher (BagMixer® 400 W, Interscience), collected in 50-mL tubes, and then centrifuged for 10 min at 20,089*g*. The juice components were then analyzed using a WineScanTM Auto based on Fourier Transform Infrared Spectroscopy (FTIR; FOSS Analytical, Hillerød) (Destrac et al., 2015; Suter et al., 2021). Sugar content represented by total soluble solids (°Bx) was measured with a digital refractometer.

Vine water status was determined from the juice extracted from the berries by measuring the δ^13^C, which refers to the ratio of carbon isotopes ^13^C/^12^C. The δ^13^C (in ‰) varies between −20‰, considered as severe water stress, and −28‰, considered as no water stress (Van Leeuwen et al., 2009).

Approximately 20 leaves were sampled in triplicate for each area and dried at 40°C for 48 h. Blades and petioles were separated and sent to Auréa Agrosciences (Blanquefort, France) to measure the Mg, Ca, K, C, P, and N content.

### 2.3 Soil, rhizosphere and root sampling

A schematic summary of the samplings and measurements conducted during this study is depicted in **Supplementary Figure S2**. All soil and root samples were taken in May 2020. In each area, five mature grapevine plants (>10 years old) were randomly selected for further analyses. Approximately 500 g of bulk soil samples per area were collected at depths of 10 to 30 cm from the inter-row close to these five selected vines using an auger (10 cm × 25 cm).

Approximately 5 g of whitish juvenile roots with soil attached to them were collected in tubes containing sterile 0.85% NaCl solution. Soil aggregates were removed from the roots by manual shaking, and the proximal soil attached to the roots was considered as rhizosphere soil. The rhizosphere was separated from roots using centrifugation at 2,000*g* subsequently to 10 s of vortex and repeated twice. At this stage, the roots were treated separately and subdivided into two groups: one for the estimation of mycorrhizal colonization, and the other for microbial endosphere analysis by amplicon sequencing. The roots in this latter group were surface sterilized by adding 3% sodium hypochlorite for 1 min, followed by 3% H_2_O_2_ for 1 min. The roots were washed three times with sterile water and stored at −80°C until DNA extraction.

Each rhizosphere and bulk soil sample was subdivided into two groups: one for cultivable microbial analysis and the other for quantitative PCR (q-PCR) and amplicon sequencing. The latter group was lyophilized for 48 h using Christ Alpha® 1–4 (Bioblock Scientific) and stored at −80°C prior to DNA extraction.

In addition, pedological profiles were performed in spring 2021 in both S and AS areas using a mini excavator, distinguishing three horizons in the S area and four horizons in the AS area (**Supplementary Figure S2B**). Samples for each horizon were taken in triplicate for analysis, consisting in physicochemical parameter assessment, enzymatic assays, DNA extraction, cultivable bacteria and fungi coupled to Eco-Plates measurements, and q-PCR.

### 2.4 Physicochemical parameters and enzymatic assays of bulk compartment and deep soil horizons

Enzymatic assays (arylamidase, β-glucosidase, and phosphatase) were carried out as described in Darriaut et al. (2021), with fresh, homogenized, and sieved samples of the five bulk soil samples from each area. One gram of fresh soil from each site was dried and weighed for the final enzymatic activity calculation. Approximately 450 g of each of the five bulk samples were used to create a pool (the remaining bulk samples were treated individually for the rest of the analysis), and 500 g of this pooled soil were sampled in triplicate, dried at 40°C for 72 h, sieved (<2 mm), and sent to INRAe LAS (62000, Arras) for physicochemical measurements including basic soil properties (i.e., grain size, pH, CEC, C, and N content), micro and macronutrients (i.e., organic matter, P, Ca, Mg, K, Na, NO_3_^−^, and NH_3_-N), and trace elements (i.e., Cu, Fe, Mn, and Zn).

### 2.5 Potential metabolic diversity, quantification of cultivable microorganisms in rhizosphere and bulk soil, and mycorrhizal root colonization analysis

Potential metabolic diversity (PMD) and quantification of cultivable bacteria and fungi from fresh rhizosphere and bulk soils were carried out as described in Darriaut et al. (2021). This involved plating soil dilutions on R2A medium amended with 25 mg L^−1^ of nystatin to quantify the cultivable bacterial population while quantifying the fungal populations on PDA medium supplemented with 500 mg L^−1^ of gentamicin and 50 mg L^−1^ of chloramphenicol.

In parallel, PMD was evaluated on three individuals chosen randomly using the Biolog Eco-Plates™ system (Biolog Inc., CA), by measuring 31 different substrates (i.e., amines, amino acids, carbohydrates, carboxylic acids, phenolic compounds, and polymers) consumed by microorganisms present, every 24 h for 4 days.

From the subgroup of fresh root samples that were not surface sterilized, 30 subsamples of fresh roots were used to evaluate their colonization by mycorrhizal fungi. These roots were stained using the modified ink-KOH-H_2_O_2_ method (Phillips and Hayman, 1970), and arbuscular mycorrhizal colonization was estimated as described in Darriaut et al. (2022a). Briefly, the samples were rinsed in sterile water, incubated in 10% KOH for 30 min at 95°C, and depigmented with 3% H_2_O_2_. After three successive washes with sterile water, the samples were colored by incubation at 90°C for 5 min using a mixture of 5% India ink (Super Black™) and 8% acetic acid solution. Samples were finally destained with 8% acetic acid for 15 min at room temperature before being observed (*n* = 30, 1 cm). The arbuscular mycorrhizal colonization was estimated with the Trouvelot et al. (1986) method and Mycocalc (https://www2.dijon.inrae.fr/mychintec/Mycocalc-prg/download.html).

### 2.6 DNA extraction

DNA was extracted from 250 mg of the lyophilized soils using the DNeasy PowerSoil Pro kit (Qiagen) according to the manufacturer recommendations with an additional C5 washing step. Soil DNA extraction was initiated using a FastPrep device set at power level 4 m s^−1^ for 30 s and performed twice with a vortex step between each run.

Surface-sterilized root samples stored at −80°C were ground by bead beating in steel containers using Retsch MM400 with liquid nitrogen. DNA was extracted from 100 mg of root powder using the DNeasy® Plant Mini kit, following the manufacturer’s instructions.

DNA samples were quantified on a Qubit® 3.0 fluorometer (Thermo Fisher Scientific) using the Qubit™ dsDNA HS Assay kit, and their quality was checked with a NanoDrop™ 2000/2000c spectrophotometer (Thermo Fisher Scientific).

### 2.7 Quantitative PCR amplification of bacterial, archaeal, and fungal genes

q-PCR analyses were performed on the DNA extracted from the soil samples using three primers pairs to quantify bacterial and archaeal 16S rRNA genes as well as the fungal and mycorrhizal 18S rRNA genes (**Supplementary Table S1**), according to Darriaut et al. (2021). Briefly, q-PCR based on absolute quantification was performed on archaeal (Arch967F/Arch1060R), bacterial (341F/515R), and fungal (FF390/FR1) communities. The genes used as standards were subcloned using the pGEM®-T easy vector system (Promega). The q-PCR analyses were performed in three replicates per samples using the GoTaq® q-PCR Master Mix (Promega).

### 2.8 Pre-processing of 16S gene and ITS sequencing and bioinformatic analysis

The DNA samples were randomized across plates and amplified using the universal primers, including the specific overhang Illumina adapters listed in **Supplementary Table S1**, specific to either the bacterial and archaeal 16S rRNA gene (785R/341F), or the fungal ITS1 region (ITS1F/ITS2).

Each of the 25-μL reaction contained 5 μL of 5X GoTaq® Reaction Buffers (Promega, France), 15.875 μL of Nuclease-free water, 0.5 µL of mixed dNTPs (10 mM), 0.5 μL of each primer (10 μM), 2.5 μL of DNA template (5 ng/μL), and 0.125 µL of GoTaq® G2 DNA Polymerase (5 U/µL) (Promega, France).

PCR amplifications were performed in five replicates for each gene. The cycling conditions of 16S rRNA gene differed from the ITS amplification, which were initiated with denaturation at 95°C for 5 min, followed by 25 and 30 cycles, respectively, with denaturation at 95°C for 30 s, an annealing step at 55°C for 30 s, followed by an extension step at 72°C for 30 s and 45 s, respectively. Further steps were carried out at the PGTB sequencing facility (Genome Transcriptome Facility of Bordeaux, Pierroton, France) using a V2 with 2 × 250 nucleotide paired reads protocol. The PCR products were purified with platform-specific SPRI magnetic beads (1× ratio) and quantified using the Quant-iT™ dsDNA Assay kit (ThermoFisher, France). MID and Illumina sequencing adapters were added. Libraries were pooled in equimolar amounts using a Hamilton Microlab STAR robot and sequenced on an Illumina MiSeq platform using the MiSeq Reagent Kit V2 (2 × 250 bp). Obtained sequences were demultiplexed with index search at the PGTB facility.

The quality of the obtained sequences were first checked with FastQC v.0.11.8 (Andrews, 2010). Sequences were quality filtered, trimmed, denoised, and clustered into Operational Taxonomy Units (OTUs) using the FROGS pipeline from Galaxy instance (Escudié et al., 2018). This involved assembling raw forward and reverse reads for each sample into paired-end reads with a minimum overlapping of 50 nucleotides and 0.1 mismatch using the VSEARCH tool (Rognes et al., 2016). Primers were removed using Cutadapt (Martin, 2011), chimeras were detected and removed with UCHIME (Edgar et al., 2011), and clustering was performed using SWARM (Mahé et al., 2014) in the FROGS pipeline. The minimum sequence abundance proportion was set at 5e^−5^ to keep OTUs. Taxonomic assignments of 16S rRNA and ITS-based OTUs were performed against silva138.1 (16S pintail100) and Unite8.2, respectively, using Blast from Galaxy. Datasets were gathered and analyzed via *phyloseq* (1.38.0). Taxa related to mitochondrial and chloroplast OTUs were removed using the Arabido_TAIR10_Chl_Mito databank.

### 2.9 Functional inferences of bacterial and fungal communities

Phylogenetic investigation of communities by reconstruction of unobserved states (PICRUSt) was used to predict the functional composition of the bacterial 16S rRNA marker (Douglas et al., 2020). PICRUSt2, which relies on OTUs, was run on the pipeline integrated within the Galaxy instance. SEPP placement tool was used to insert the sequences into the reference tree with a minimum alignment length setting of 0.8. The hidden-state prediction with maximum parsimony method was used to predict the function abundances with the KO (KEGG pathway) database The Nearest Sequenced Taxon Index (NSTI) cutoff was set to 0.5, excluding 976 clusters and keeping 1,412 clusters. Only the classifications related to “Metabolism” and “Environmental Information Processing” were kept for statistical analyses, with their relative abundances assessed as a percentage of the total abundances.

The “funguild_assign” function from the *FUNGuildR* (0.2.0.9000) package was used with the FUNGuild database to taxonomically parse trophic modes and guilds of functional traits among fungal communities, based on OTUs. Only the guild confidences classified as “Highly probable” and “Probable” were selected for statistical analyses. OTUs assigned to more than two trophic modes (i.e., pathotroph, saprotroph, and symbiotroph) or more than two guilds (i.e., wood saprotroph, undefined saprotroph, plant saprotroph, orchid mycorrhizal, lichenized, epiphyte, plant pathogen, fungal parasite, endophyte, ectomycorrhizal, arbuscular mycorrhizal, and animal pathogen) were classified as “Multi-affiliated”.

### 2.10 Statistical analyses

All analyses and graphs were performed on R (R-4.2.1) using RStudio (2021.9.1.372). The figures were generated with *ggplot2* (3.4.0) and *ggthemes* (4.2.4) packages and arranged using *ggpubr* (0.5.0). Two-way analysis of variance (ANOVA) with soil status (AS or S) and depth of horizons in the pit (four horizons in AS and three horizons in S areas) factors were performed on enzymatic activities, cultivable, q-PCR, Eco-Plates measurements, and abundances of functional OTUs. Residuals were checked for independence, normality, and variance homogeneity using Durbin–Watson, Shapiro–Wilk, and Bartlett tests, respectively. When assumptions for parametric tests were not respected, a multiple pairwise comparison was performed using a Wilcoxon test subsequently to a Kruskal–Wallis test using the *multcomp* (1.4-20) package. Principal component analysis (PCA) was performed using *FactoMineR* (2.7) and *missMDA* (1.18). Area under the curve (AUC) of average color well development (AWCD), which gives better insights for curve dynamics, was calculated with the trapezoidal method for each soil using *caTools* (1.18.2).

For amplicon analyses, shared OTUs were visualized with Venn diagrams generated with *VennDiagram* (1.7.3). Richness and α-diversity metrics, represented by Chao1, Simpson’s diversity, and Bray–Curtis dissimilarity, respectively, were calculated via *phyloseq* (1.38.0) using “estimate_richness” and “distance” functions. Pairwise comparisons were performed to test for significant differences between the means of alpha diversity metrics by conditions, based on either *t*-tests or Wilcoxon tests, subsequently to homogeneity and normalization verifications using Levene and Shapiro tests. Nonmetric multidimensional scaling (NMDS) was used to “ordinate” samples in two-dimensional space based on Bray–Curtis distance using ordinate function from *phyloseq* with the NMDS method. Permutation multivariate analysis of dispersion was conducted with the “betadisper” function from *vegan* (2.6-4) to test the homogeneity of multivariate dispersions among soil status (i.e., S and AS) or compartments (i.e., bulk, rhizosphere, and roots). Linear models and permutational multivariate analysis of variance (PERMANOVA) were demonstrated for richness and diversities metrics, using the following formula: variable ~ Soil status × Compartment. Type II ANOVAs were performed using *car* (3.0-12) on Chao1 and Simpson’s diversity metrics while PERMANOVAs were assessed on Bray–Curtis dissimilarity using the “adonis2” function from *vegan* with 999 permutations. The “ggeffectsize” and “ggdiffbox” functions from *MicrobiotaProcess* (1.6.2) were used to discriminate significantly different taxa across conditions. This process was set with a Kruskal (α = 0.05) test based on linear discriminant analysis (LDA), effect size (LEfSe), and Wilcox (α = 0.05), corrected with false discovery rate (FDR). MicrobiomeMarker (version 1.1.1) was used for Limma-Voom method, using “run_limma_voom” function (α = 0.05) corrected with the FDR to discriminate significantly enriched taxa above genus level in either the symptomatic or asymptomatic conditions in root and rhizosphere compartments. Pearson’s correlation analysis was performed, using “cor.test” from *stats*, between the utilization of the families of carbon sources from Eco-Plates measurements and functional KEGG pathways for microbial communities, as well as the top 10 bacterial and fungal genera.

Partial least squares path modeling (PLS-PM) was elaborated using *plspm* (0.5.0) to integrate the convoluted interrelationships between 13 latent variables based on soil status, bulk, rhizosphere, and root microbial communities, as well as leaf and berry composition. After a first run of the model, variables with loadings less than 0.7 (i.e., correlations between the latent variable and its indicators) were removed. The model reliability was evaluated using the goodness of fit (GoF) and the determination coefficient (*R*²), which are used to conclude that the structural model is valid and of good quality when *R*² > 0.6 and GoF > 0.7. The exhaustive understanding of the direct and cross-loading effects of these latent variables was also evaluated to further explain the PLS-PM.

## 3 Results

### 3.1 Declining vines showed altered biochemical composition of berries and petioles compared to healthy vines

**Figure 1:**
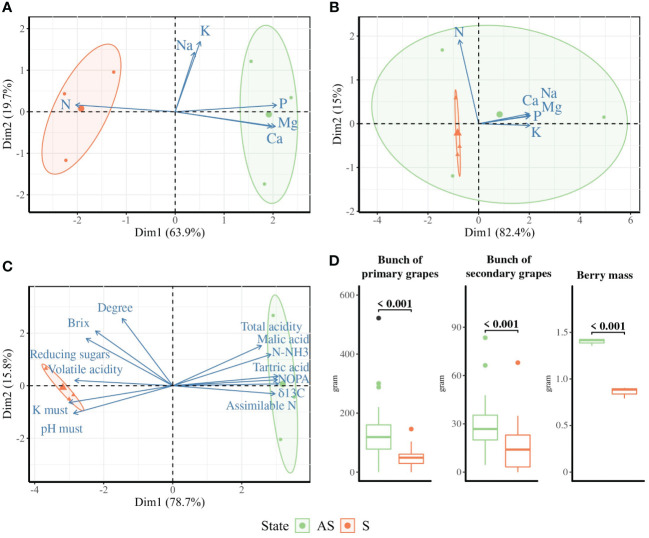
Ordination biplot PCA for **(A)** petiole, **(B)** leaf blade, and **(C)** must compositions across symptomatic (orange, S) and asymptomatic (green, AS) areas. The size of the arrows indicates the contribution strength of the variables. Standard error ellipses show 95% confidence areas. **(D)** Yield is presented as the mean mass of primary and secondary bunches per plant (*n* = 28) and the average berry mass over 100 berries (*n* = 3). *p*-values were calculated using *t*-tests or Wilcoxon tests depending on the parametric assumption.

The biplot analysis of petiole (**Figure 1A**) and must (**Figure 1C**) compositions revealed distinct profiles across symptomatic S and asymptomatic AS areas, with dimensions accounting for 83.6% and 94.5% of total variance, respectively.

The leaf blade composition profile of the AS area overlapped that of the S leaf blade profile (**Figure 1B**), with dimensions accounting for 97.4%. In the biplot PCA of petiole biochemical parameters, AS samples appear on the positive side of Dim1, which correlated with phosphorus (P), magnesium (Mg), calcium (Ca), potassium (K), and sodium (Na), while nitrogen (N) is found on the negative side of Dim1, where all the S samples are represented. Even if a significantly lower amount of P, Ca, and Mg and a higher content of N was found in petioles from S area, the nutritional status seems correct for both areas (**Supplementary Table S2**).

In the must, the content of alpha-amino N (NOPA), assimilable N, ammoniacal N (N-NH_3_), total acidity, malic acid, tartaric acid, and δ^13^C correlated with the positive side of Dim1, where the asymptomatic samples were found. The K, pH, volatile acidity, reducing sugars, brix content, and degree were in correlation with the negative side of Dim1, which displayed all the symptomatic samples (**Figure 1C**). The results highlighted significantly less malic acid, tartaric acid, and content in assimilable N, NOPA, and N-NH_3_ and significantly higher pH and potassium in must composition from the S area compared to the AS area (**Supplementary Table S2**). The vine water status represented here by δ^13^C was not significantly different between S and AS areas and did not reflect a severe water deficit.

During harvest time in autumn, as expected, the berry mass and yield, represented here by the total biomass of primary and secondary bunches and number of harvested grapes, were significantly higher in the AS area compared to the S area (**Figure 1D**).

### 3.2 Microbial activity in deep horizons does not appear to be correlated with grapevine decline

Two pits were dug, one in the S area and the other in the AS are, with a depth of 120 and 140 cm, respectively (**Supplementary Figure S2B**). In the S area, macroscopic visual observation such as root structure and soil compactness could not explain the low vigor (Darriaut et al., 2021) and yield of the plants (**Supplementary Table S3**). Each of the detected soil horizons displayed distinct physicochemical features and were classified as sand, except for latter depths, which were classified as loamy sand and sandy clay loam in S and AS pits, respectively. The physicochemical parameters were measured for each horizon of the two areas and are reported in **Supplementary Table S3**, with higher CEC, N, C, C/N, OM, and nitrate content in the first two horizons of the AS area compared to the S area.

Various measurements were taken to assess the microbiological state of the different soil horizons (**Table 1**).

**Table 1:** Measurements made at different depths across the pits in both S and AS areas.

	Depth (cm)	S	Depth (cm)	AS
Bacterial counts Log (CFUs g^−1^_dry soil_)	0–25	6.47 ± 0.03 ad	0–25	6.54 ± 0.02 a
	25–50	6.45 ± 0.02 d	25–60	6.19 ± 0.06 b
	50–120	5.32 ± 0.11 e	60–95	5.87 ± 0.09 c
			95–140	5.69 ± 0.11 c
Fungal counts Log (CFUs g^−1^_dry soil_)	0–25	4.43 ± 0.02 e	0–25	4.90 ± 0.10 a
	25–50	3.71 ± 0.04 f	25–60	4.20 ± 0.03 b
	50–120	2.75 ± 0.07 d	60–95	3.95 ± 0.01 c
			95–140	2.94 ± 0.05 d
Arylamidase activity (µg_2-naphthylamine_ g^−1^_dry soil_ h^−1^)	0–25	65.26 ± 0.57 e	0–25	183.20 ± 1.72 a
	25–50	5.77 ± 0.23 d	25–60	59.87 ± 0.39 b
	50–120	4.23 ± 0.23 f	60–95	12.18 ± 0.35 c
			95–140	5.13 ± 0.23 d
β-glucosidase activity (µg_p-nitrophenol_ g^−1^_dry soil_ h^−1^)	0–25	12.61 ± 0.73 d	0–25	32.16 ± 1.81 a
	25–50	5.20 ± 0.27 c	25–60	8.15 ± 0.25 b
	50–120	1.20 ± 0.66 d	60–95	5.66 ± 0.41 c
			95–140	3.64 ± 1.36 cd
Phosphatase activity (µg_p-nitrophenol_ g^−1^_dry soil_ h^−1^)	0–25	53.42 ± 4.35 a	0–25	50.66 ± 3.28 a
	25–50	65.94 ± 7.52 a	25–60	43.23 ± 3.85 a
	50–120	129.57 ± 6.94 d	60–95	17.72 ± 2.43 b
			95–140	5.03 ± 1.92 c
Total DNA extracted (µg g^−1^_dry soil_)	0–25	29.23 ± 2.36 d	0–25	61.44 ± 1.85 a
	25–50	5.29 ± 0.78 c	25–60	20.61 ± 0.93 b
	50–120	1.63 ± 0.26 c	60–95	4.86 ± 0.70 c
			95–140	1.75 ± 0.30 c
Archaeal 16S (10^8^ number of copies)	0–25	0.62 ± 0.03 b	0–25	1.88 ± 0.32 a
	25–50	0.0009 ± 0.0007 c	25–60	0.42 ± 0.09 b
	50–120	0.0002 ± 0.00005 c	60–95	0.038 ± 0.004 c
			95–140	0.00018 ± 0.00005 c
Bacterial 16S (10^9^ number of copies)	0–25	0.46 ± 0.01 d	0–25	1.51 ± 0.15 a
	25–50	0.0007 ± 0.00002 c	25–60	0.25 ± 0.04 b
	50–120	0.0003 ± 0.00009 c	60–95	0.024 ± 0.007 c
			95–140	0.00021 ± 0.00002 c
Fungal 18S (10^7^ number of copies)	0–25	0.352 ± 0.009 d	0–25	1.58 ± 0.35 a
	25–50	0.0012 ± 0.0006 c	25–60	0.09 ± 0.02 b
	50–120	0.00027 ± 0.00018 c	60–95	0.008 ± 0.004 c
			95–140	0.00009 ± 0.00002 c

Numbers represent means ± SE (n = 3), and letters indicate different groups obtained for each variable after pairwise comparisons with Bonferroni correction.

Microbial counts, DNA indicators, and enzymatic activities decreased with soil depth in both areas surveyed, with the exception of alkaline phosphatase activity in the soil in the S area, which presents an inverted profile.

Significant differences were detected between the horizons of the two areas, mainly in the first two horizons below the surface, with higher values in the AS area for all parameters except alkaline phosphatase activity (**Table 1**; **Supplementary Figure S3**).

The biplot PCA also revealed a distinct profile for both S and AS (**Supplementary Figure S3A**), with symptomatic features explained by alkaline phosphatase activity. As described above, the deeper the horizon in the S pit, the higher the phosphatase activity, whereas other enzymatic activities, arylamidase and β-glucosidase, drastically drop off after the second horizon in both areas (**Supplementary Figure S3B**).

### 3.3 Bulk soils showed similar physicochemical properties but had contrasting enzymatic and microbial profiles

**Figure 2:**
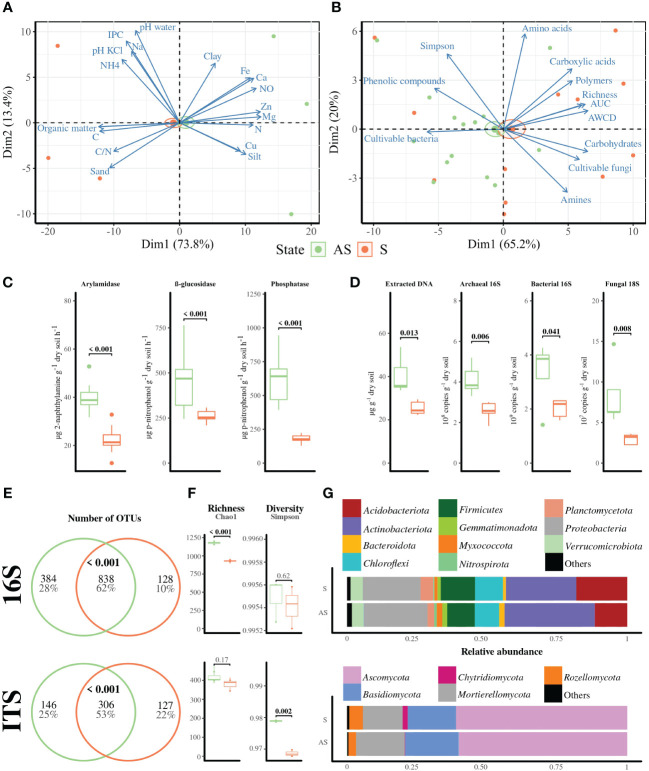
Bulk soil profile in symptomatic (orange, S) and asymptomatic (green, AS) areas. Biplot PCA of **(A)** physicochemical parameters (*n* = 3) and **(B)** Eco-Plates measurements represented by Simpson’s index, AWCD, AUC, functional richness, and family compounds consumed (i.e., amines, amino acids, carbohydrates, carboxylic acids, phenolic compounds, and polymers) coupled to bacterial and fungal level of cultivable populations. Standard error ellipses show 95% confidence areas. **(C)** Enzymatic activities represented by arylamidase, β-glucosidase, and phosphatase alkaline, and **(D)** q-PCR measurements for archaeal and bacterial 16S and 18S rRNA genes (*n* = 5). **(E)** Shared OTUs represented by Venn diagram with significant overlaps detected using hyper-geometric tests, and **(F)** α-diversity metrics (richness = Chao1, diversity = Simpson), as well as **(G)** relative abundance of phyla. Significant differences, except for shared OTUs, were calculated with *t* or Wilcoxon tests, depending on the normality hypothesis.

As the most significant differences were found in the superficial horizon, we focused further analyses on bulk soils (10–30 cm depth) harvested near the five selected vines for each area. The biplot PCA for physicochemical parameters in bulk soils revealed an overlap of confidence intervals in the S and AS profiles, with dimensions accounting for 87.2% of total variance (**Figure 2A**). Although CEC, nitrogen, calcium, NO_3_^−^, and manganese content were significantly higher, whereas carbon/nitrogen ratio was significantly lower in AS compared to S bulk soil, the observed differences did not fall within a range that will likely explain differential vine growth (**Supplementary Table S4**).

The biplot analysis related to Eco-Plates measurements, with Dim1 and Dim 2 accounting for 86.8% of total variance, also revealed an overlap with most of the variables explaining the symptomatic feature (**Figure 2B**). The level of cultivable bacterial populations was significantly higher and the fungal level was significantly lower in AS compared to S bulk soil (**Supplementary Table S5**), while no significant differences were found in Eco-Plates measurements except for AUC, which was significantly higher in the S area than in the AS area. However, enzymatic activities involved in nitrogen, carbon, and phosphorus cycling (i.e., arylamidase, β-glucosidase, and alkaline phosphatase, respectively) were significantly higher in AS compared to S bulk soil (**Figure 2C**). Similarly, total molecular biomass, copies of fungal 18S rRNA genes, and bacterial and archaeal 16S rRNA genes were significantly higher in AS compared to S bulk soils (**Figure 2D**).

A total of 1,111,642 raw sequences were generated for the amplicon-based sequencing, irrespective of the compartment. After chimera removal, paired-end sequences of 16S rRNA gene and ITS were clustered into 1,566 and 961 operational taxonomic units (OTUs), respectively. The OTU accumulation curves tended toward saturation as the number of samples increased, indicating that the sequencing depth was sufficient to provide an overview of the taxonomic distribution of each microbial community in the samples (**Supplementary Figure S4**).

Approximately 62% and 53% of the bacterial 16S rRNA and fungal ITS OTUs, respectively, were shared between S and AS bulk soils (**Figure 2E**). Richness, represented here by the Chao1 metrics, was similar for fungal communities but significantly higher in AS compared to S bulk soil for the bacterial OTUs (**Figure 2F**). On other hand, diversity, represented here by Simpson’s index, was similar for bacterial communities, while the asymptomatic bulk soil presented higher fungal diversity than the symptomatic one.

*Actinobacteriota* (29%), *Proteobacteria* (22%), *Acidobacteria* (15%), *Firmicutes* (11%), *Chloforexi* (9%), *Verrucomicrobiota* (5%), Plancomycetota (3%), Gemmatimonodata (2%), Bacteroidota (1%), Myxococcota (1%), and Nitrospirota (1%) were the most abundant bacterial phyla of bacterial communities (**Figure 2G**), while the “Others” (<1%) group was composed of *Dependentiae*, *Desulfobacterota*, *GAL 15*, *Latescibacterota*, *Methylomirabilota*, and *RCP2-25*, *WPS-2* phyla. Regarding the ITS-based sequences, *Ascomycota* (65%), *Basidiomycota* (17%), *Mortierellomycota* (14%), *Rozellomycota* (5%), and *Chytridiomycota* (1%) were the predominant phyla, while *Blastocladiomycota*, *Calcarisporiellomycota*, *Entorrhizomycota*, *Glomeromycota*, *Kickxellomycota*, *Olpidiomycota*, and *Zoopagomycota* were grouped to “Others” (<1%).

### 3.4 Rhizosphere and root-associated microorganisms differed between symptomatic and asymptomatic conditions

The biplot PCA of Eco-Plates measurements and the level of cultivable bacteria and fungi from rhizosphere samples accounted for 97.4% of total variance. This analysis revealed overlapping profiles between S and AS areas, with Eco-Plates measurements explaining the symptomatic features (**Supplementary Figure S5A**). Interestingly, the global AWCD represented by AUC was significantly higher in S rhizosphere than AS, specifically with significantly more carbohydrates, carboxylic acids, phenolic compounds, and polymers consumed after 96 h of Eco-Plates incubation (**Supplementary Table S5**). Regarding the q-PCR assays, only bacterial 16S rRNA gene was significantly more important in AS rhizosphere compared to the S rhizosphere (**Supplementary Figure S5B**).

For amplicon-based sequencing, 11.2% of the total bacterial OTUs were shared between the four conditions (i.e., symptomatic and asymptomatic root, and rhizosphere) (**Supplementary Figure S6A**). In the rhizosphere, 82% of the assigned bacterial sequences and 67.3% of the assigned fungal sequences were common to S and AS, regardless of the root compartment. Similarly, in the roots, regardless of the rhizosphere compartment, 55.1% of the bacterial OTUs and 37.3% of the fungal OTUs were shared between S and AS conditions. Both bacterial and fungal richness were similar between AS and S conditions in the root endosphere samples, while significantly higher richness was found in the asymptomatic area in the rhizosphere compartment (**Supplementary Figure S6B**). There was no significant difference in terms of diversity between the S and AS conditions across the root and rhizosphere samples. The observed phyla in roots and rhizosphere were the same as those observed in bulk soil (**Figure 2G**; **Supplementary Figure S6B**).

ITS-based sequencing did not detect any significant difference [Student *t* (7.801) = 0.596, *p* < 0.568] in *Glomeromycota* phylum between AS (0.19 ± 0.06) and S (0.13 ± 0.07) roots (**Supplementary Figure S6B**). However, root staining and microscopy revealed significantly higher mycorrhizal intensity in the AS roots compared to S roots (**Supplementary Figure S7**).

**Figure 3:**
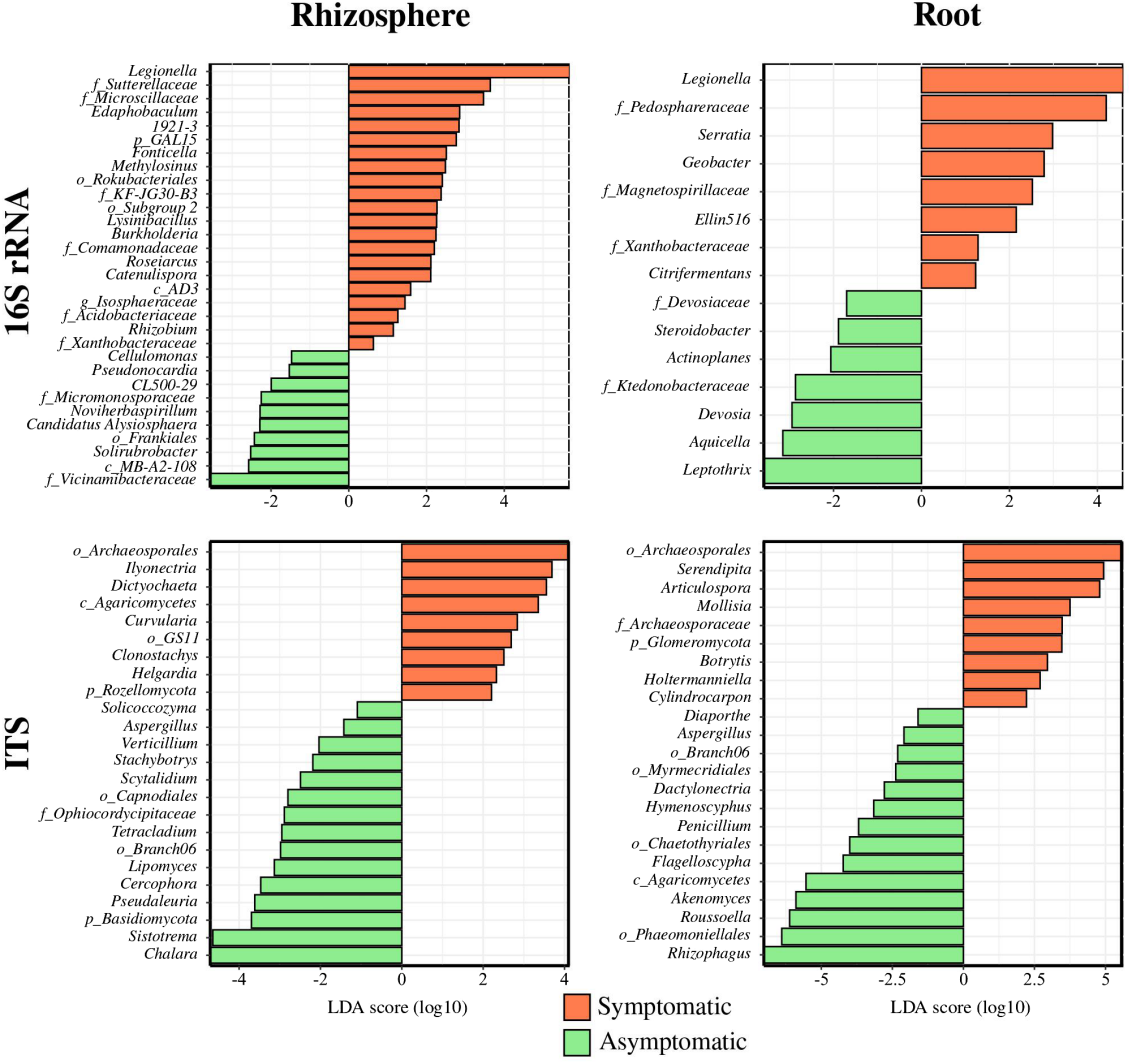
Enriched bacterial and fungal genera in symptomatic and asymptomatic root and rhizosphere compartments. Enriched bacterial and fungal genera using Limma-Voom differential analysis (*p* < 0.05) corrected with FDR are shown.

Limma-Voom differential analysis was performed to get a better overview of the differences occurring between the symptomatic and asymptomatic conditions among the bacterial and fungal communities from the root endosphere and rhizosphere compartments (**Figure 3**). This analysis detected 21 enriched bacterial groups in symptomatic rhizosphere (0.63 to 5.68 log2 fold change), mainly composed of *Proteobacteria* (*Legionella*, *Methylosinus*, *Burkholderia*, *Roseiarcus*, and *Rhizobium*) while asymptomatic rhizosphere was enriched with 10 groups (−1.47 to −3.56 log2 fold change) with a majority of *Actinobacteriota* (*Cellulomonas*, *Pseudonocardia*, *CL500-29*, and *Solirubrobacter*). In terms of fungi, nine enriched genera were found in symptomatic rhizosphere (2.20 to 4.13 log2 fold change), representing a majority of *Ascomycota* (*Ilyonectria*, *Dictyochaeta*, *Curvularia*, *Clonostachys*, and *Helgardia*), while 15 enriched taxa above the genus level were detected in AS soil (−1.50 to −7.76 log2 fold change) also belonging largely to *Ascomycota* (*Aspergillus*, *Verticillium*, *Stachybotrys*, *Scytalidium*, *Tetracladium*, *Lipomyces*, *Cercophora*, *Pseudaleuria*, and *Chalara*).

Regarding the root endosphere compartment, eight enriched genera were discriminated in the symptomatic condition (1.23 to 4.59 log2 fold change), mainly composed of *Desulfobacterota* (*Citrifermentans* and *Geobacter*) while seven taxa were found on the asymptomatic condition (−1.70 to −3.58 log2 fold change), predominantly consisting of *Proteobacteria* (*Steroidobacter*, *Devosia*, *Aquicella*, and *Leptothrix*). Similarly to the rhizosphere, the root endosphere conditions were differentially made up with *Ascomycota* with 9 genera significantly enriched in the symptomatic status (2.22 to 5.63 log2 fold change) such as *Articulospora*, *Mollisia*, *Botrytis*, and *Cylindrocarpon*, while 14 genera were enriched in the asymptomatic root (−1.60 to −7.02) including, for instance, *Diaporthe*, *Aspergillus*, *Dactylonectria*, *Hymenoscyphus*, *Penicillium*, and *Roussoella*.

### 3.5 Effect of compartmentalization on the taxonomy and the predicted functionality of microbial communities

A higher number of 16S rRNA and 18S rRNA gene copies were found in rhizosphere compared to bulk soil (**Supplementary Figure S8A**), with the same trend observed for the level of cultivable bacteria and fungi and Eco-Plates metrics (**Supplementary Figure S8B**).

**Figure 4:**
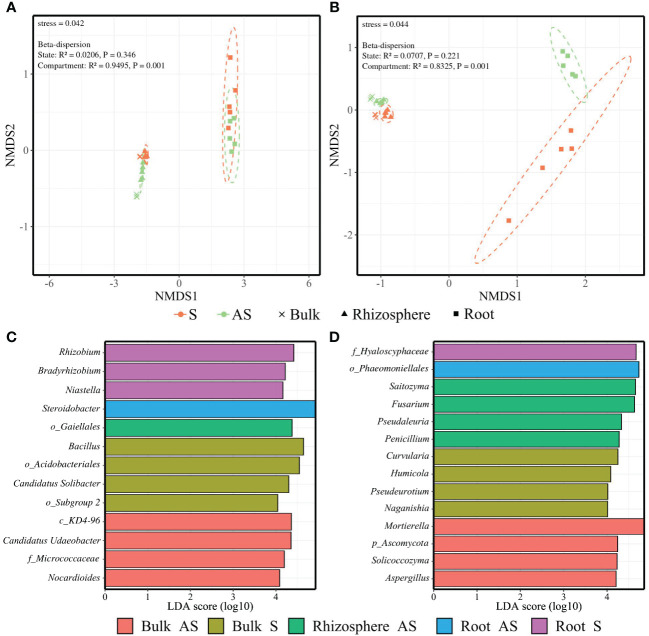
Compartmentalization effect on microbial communities. NMDS plot ordination of **(A)** bacterial and **(B)** fungal communities among the bulk (cross), rhizosphere (triangle), and root endosphere (square) across symptomatic S (orange) and asymptomatic AS (light green) soils. Dashed lines represent 95% confidence ellipses. The stress value and the results from betadisper analyses are included. LEfSe analysis (*p* < 0.05, FDR, LDA > 4) of enriched genera among the soil status × compartment conditions for **(C)** bacterial and **(D)** fungal communities with p_: phylum, c_: class, o_: order, f_: family.

Regarding amplicon sequencing, compartmentalization and soil status had a significant effect on both α- and β-diversities (**Supplementary Table S6**). Soil bacterial and fungal richness and diversity were significantly higher than that of root endosphere (**Supplementary Table S7**). In addition, NMDS based on Bray–Curtis dissimilarities demonstrated that the compartment was the main factor of difference in β-diversity for both bacterial (stress = 0.042) and fungal communities (stress = 0.044) (**Supplementary Table S6**; **Figures 4A, B**).

Despite the lack of homogeneity in dispersion due to compartmentalization, the NMDS plot still showed compartment-related clustering. The beta-dispersion analysis displayed non-significant differences for the soil status factor across the microbial communities, with evident clustering linked to the soil status. The strongest relationship between the factors and bacterial or fungal communities was reported for compartment (*R*² = 0.9495 and *R*² = 0.8325, respectively) (**Figures 4A, B**).

The LEfSe (*p* < 0.05, FDR, LDA > 4) revealed nine enriched bacterial genera in bulk and rhizosphere soils mainly belonging to *Actinobacteriota* (i.e., unidentified genera from *Gaiellales*, *Micrococcaceae*, and *Nocardioides*) and *Acidobacteriota* (i.e., unidentified genera from *Acidobacteriales* and *Candidadus solibacter*) phyla, while four enriched bacterial genera found in root endosphere belonged to the *Proteobacteria* (i.e., *Rhizobium*, *Bradyrhizobium*, and *Steroidobacter*) and *Bacteroidota* (i.e., *Niastella*) phyla (**Figure 4C**). Twelve fungal genera were enriched in soil compartments belonging primarily to the *Ascomycota* (i.e., *Fusarium*, *Pseudaleuria*, *Penicillium*, *Curvularia*, *Humicola*, *Pseudeurotium*, and *Aspergillus*) and *Basidiomycota* (i.e., *Saitozyma*, *Naganishia*, and *Solicoccozyma*) phyla (**Figure 4D**). No taxa were enriched in the rhizosphere in the S area.

Moreover, fungal pathogens affiliated to grapevine diseases listed in Darriaut et al. (2023), such as gray mold, Petri disease, black foot, Phomopsis dieback, or grapevine canker, were detected in each compartment but with different abundance (**Supplementary Figure S9**). Among the 209 genera identified in the studied vineyard, *Botrytis*, *Cadophora*, *Curvularia*, *Diaporthe*, *Diplodia*, *Ilyonectria*, *Neonectria*, *Phaeoacremonium*, and *Phaeomoniella* were detected in both symptomatic and asymptomatic conditions, accounting for 1.63% of the total OTUs. A significantly higher percentage of sequences affiliated with these 10 pathogenic fungal genera, relative to the total number of sequences, was found in symptomatic compared to asymptomatic conditions in bulk (*p* < 0.001) and rhizosphere (*p* = 0.008) but not for root endosphere (*p* = 0.292). More precisely, *Botrytis*, *Cadophora*, *Curvularia*, and *Ilyonectria* were more abundant in symptomatic conditions than asymptomatic ones (**Supplementary Table S8**). Among these genera, *Botrytis caroliniana*, *Curvularia lunata*, *Curvularia spicifera*, *Curvularia portulacae*, *Curvularia inaequalis*, *Diaporthe columnaris*, *Ilyonectria destructans*, *Cadophora luteo-olivacea*, *Diplodia intermedia*, *Neonectria lugdunensis*, and *Phaeomoniella chlamydospora* were detected.

**Figure 5:**
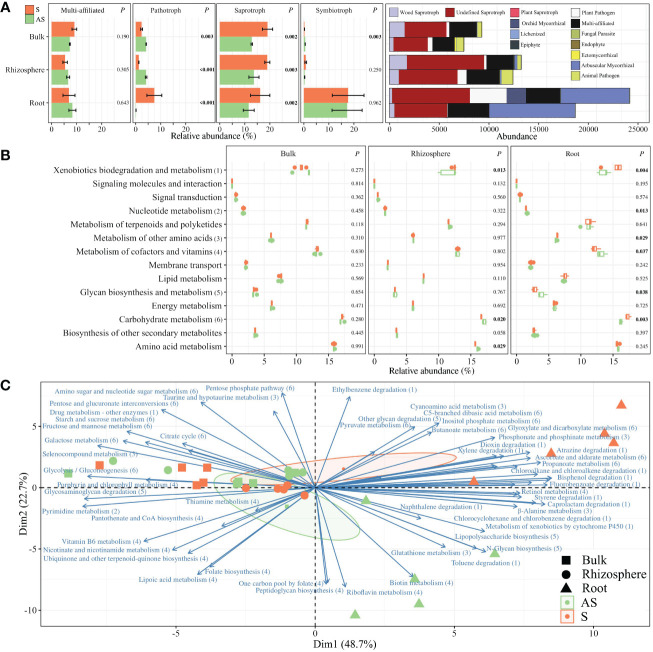
Functional inference of **(A)** fungal community using the FUNGuild database represented by the relative abundances of the trophic modes (i.e., multi-function, pathotroph, saprotroph, and symbiotroph) and the total abundances of associated guilds. Predicted **(B)** pathways and **(C)** functions of bacterial communities using PICRUSt2. Barplots are represented with means ± standard error. *p*-values were calculated using Student *t* or Wilcoxon tests. Biplot analysis represents only the functions in which pathways were significantly different between S and AS roots, represented by 1 to 6 in **(B)**.

In addition, functional prediction analysis on fungal communities was performed using FUNGuild, which displayed four trophic modes (i.e., multi-affiliation, pathotroph, saprotroph, and symbiotroph) and 13 guilds (i.e., multi-affiliation, wood saprotroph, undefined saprotroph, plant saprotroph, orchid mycorrhizal, lichenized, epiphyte, plant pathogen, fungal parasite, endophyte, ectomycorrhizal, arbuscular mycorrhizal, and animal pathogen) (**Figure 5A**). Saprotrophs were significantly enriched in S compared to AS areas for bulk, rhizosphere, and root compartments (**Figure 5A**), with increased specific abundances of wood, plant, and undefined saprotrophs (**Supplementary Figure S10**). Pathotrophs were enriched in symptomatic roots, while bulk and rhizosphere compartments were predominantly composed of pathotrophs in asymptomatic conditions. No differences were detected regarding the symbiotrophs, except in the bulk soil.

**Figure 6:**
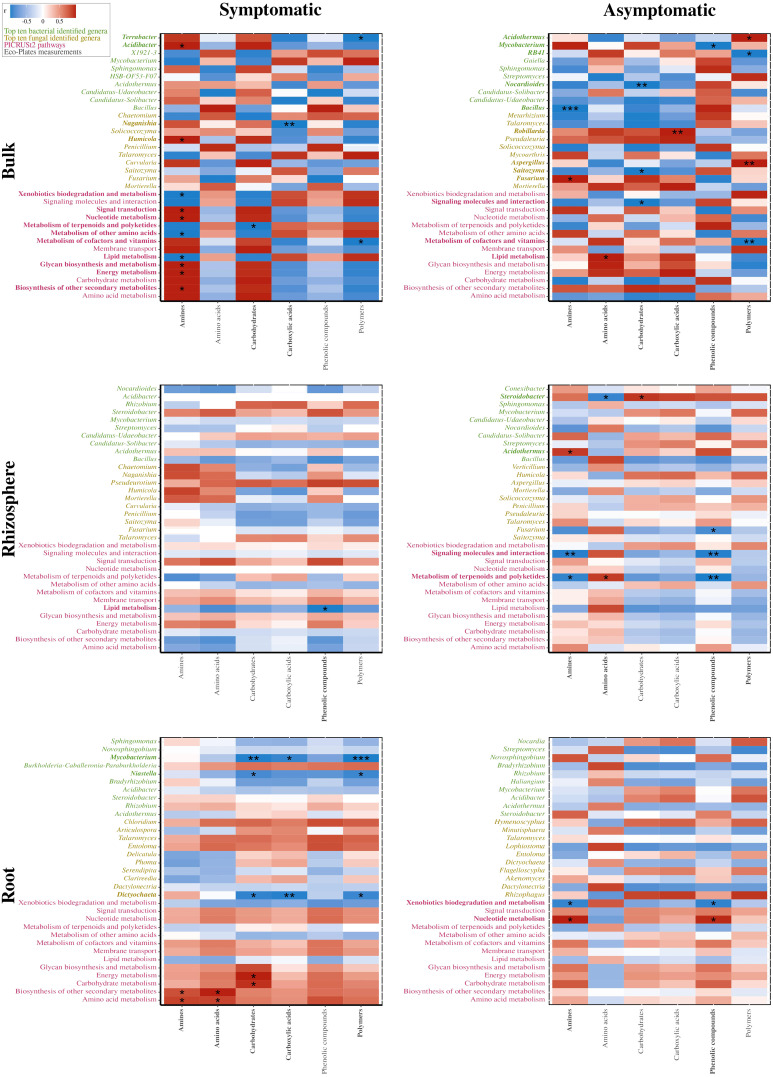
Correlation analyses between consumption of the grouped carbon sources from Eco-Plates measurements (gray) and the dominant bacterial (green) and fungal (yellow) genera, and the PICRUSt2 inferences from the microbial communities (pink) present in symptomatic (left) and asymptomatic (right) bulk (top), rhizosphere (middle), and root (bottom) compartments. For root analyses, the Eco-Plates measurements were the ones from the rhizosphere samples since no Eco-Plates assays were performed on root samples. The color intensity shows the *R*-value of correlation in each panel, and the asterisk represents significant correlations (* indicate *p*<0.05; ** *p*<0.01; *** *p*<0.001). Significantly correlated variables were presented in bold.

Furthermore, potential metabolic pathways of bacterial communities were estimated using PICRUSt2. The 1,412 predicted clusters formed 14 selected pathways among the “Metabolism” and “Environmental Information Processing” classifications (**Figure 5B**). No significant differences were detected between S and AS bulk soils, while enriched abundances of taxa linked to carbohydrates and amino acids pathways were upregulated in AS rhizosphere. Most of the significant differences were detected in roots, with enriched abundances for pathways in the S condition compared to AS, such as metabolism of xenobiotics, carbohydrates, and other amino acids whereas the metabolism of nucleotides, glycans, cofactors, and vitamins were upregulated in asymptomatic roots. Considering these six differential pathways in roots, 58 predicted functions were established and represented in a biplot analysis, which revealed distinct profiles across symptomatic and asymptomatic areas, with dimensions accounting for 71.4% of total variance (**Figure 5C**). The positive side of Dim1 was exclusively composed of root samples, divided between symptomatic conditions for the upper side of Dim2 and asymptomatic samples for the negative side of Dim2. In addition, correlation analysis demonstrated that *Mycobacterium* (*r* = −0.987; *p* = 0.002), *Niastella* (*r* = −0.894; *p* = 0.040), and *Dictyochaeta* (*r* = −0.958; *p* = 0.010) in symptomatic roots were significantly correlated with carbohydrate consumptions in Eco-Plates from symptomatic rhizosphere, which was also significantly correlated with carbohydrate metabolism (*r* = 0.999; *p* = 0.025) and energy metabolism (*r* = 0.871; *p* = 0.043) in symptomatic roots (**Figure 6**).

Analogously, in asymptomatic roots, no significant correlation was detected between fungal or bacterial genera with Eco-Plates measurements. Regarding the symptomatic bulk soil, *Acidibacter* (*r* = 0.999; *p* = 0.034) and *Humicola* (*r* = 0.998; *p* = 0.043) were significantly correlated with amines consumption from Eco-Plates, while *Bacillus* (*r* = −0.999; *p* ≤ 0.001) and *Fusarium* (*r* = 0.999; *p* = 0.016) were significantly correlated with amines substrate in asymptomatic bulk soil. Interestingly, in symptomatic roots, the predicted functions related to energy (*r* = 0.999; *p* = 0.022) and carbohydrate (*r* = 0.992; *p* = 0.019) metabolism were significantly and positively correlated with the carbohydrate substrates used in Eco-Plates. Similarly, the amino acid metabolism (*r* = 0.992; *p* = 0.039 and *r* = 0.993; *p* = 0.033) and biosynthesis of other secondary metabolites (*r* = 0.991; *p* = 0.011 and *r* = 0.999; *p* = 0.019) were positively correlated with amines and amino acids consumed in Eco-Plates, respectively.

### 3.6 Relationships between soil status, berries, leaves, and microbial composition and functioning of the root, rhizosphere, and bulk soil compartments

**Figure 7:**
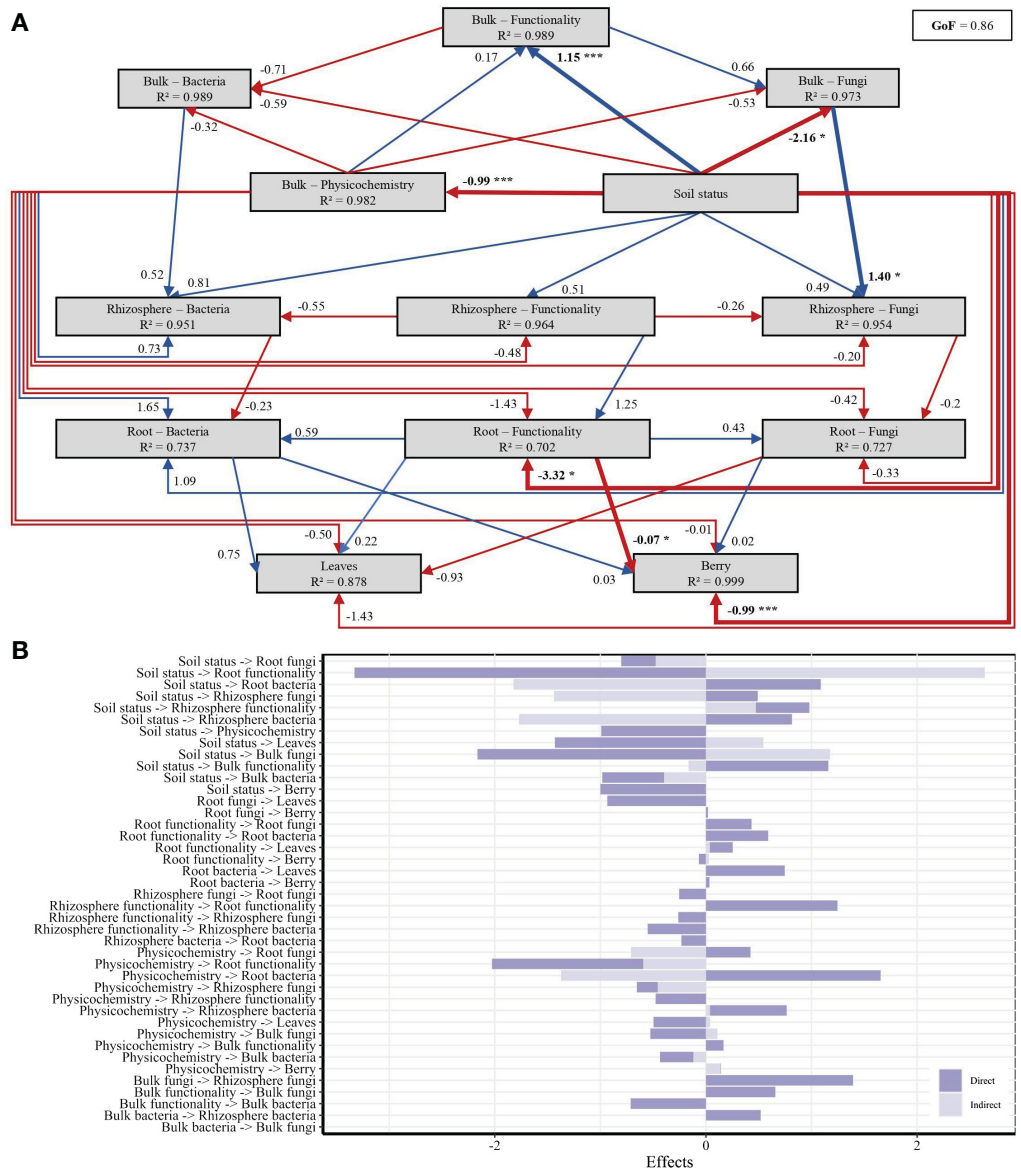
Partial Least Squares Path Modeling (PLS-PM) showing the effects of soil status on berry must, leaf composition, and microbial communities and functioning in roots, bulk soil, and rhizosphere. **(A)** PLS-PM diagram showing the positive and negative relationships, respectively represented with blue and red arrows. Asterisks indicate significant paths, *p < 0.05 and ***p < 0.001. Numbers associated with the arrows indicate standardized path coefficients. *R*² values indicate the variance of latent variables explained by the model. **(B)** Direct and indirect effects between the different latent variables tested.

A PLS-PM was done to further unravel the direct and indirect interactions between 13 latent variables (i.e., soil status, soil physicochemical properties, bulk soil functionality, bulk soil bacterial communities, bulk soil fungal communities, rhizosphere functionality, rhizosphere bacterial communities, rhizosphere fungal communities, root functionality, root bacterial communities, root fungal communities, leaves composition, and berry composition). Of the 182 variables used to construct the first PLS-PM model, 73 variables had an absolute loading value below 0.7 and were removed from the model. The variables associated with each latent variable used for the first testing model and the presented model are listed in **Supplementary Table S9**. The model, considered to be valid for expressing the complex interrelationships among the 13 latent variables (GoF ≥ 0.7; *R*² ≥ 0.6), showed that the higher positive and negative pathway coefficients were for the physicochemical parameters to communities of root bacteria pathway (*R*² = 1.65) and soil status to root microbial functionality pathway (*R*² = −3.32), respectively (**Figure 7A**).

From this PLS-PM test, it was also demonstrated that soil status was positively correlated to bulk soil functionality (direct; *R*² = 1.15; *p* = 0.006) and negatively correlated to bulk physicochemical parameters (direct; *R*² = −0.99; *p* < 0.001), bulk fungal communities (direct; *R*² = −2.16; *p* = 0.015), and berry must (direct; *R*² = −0.99; *p* = 0.002) (**Figures 7A, B**). In addition, the results indicate that soil status has a direct significant effect on root functionality (*R*² = −3.32; *p* = 0.016), based here on PICRUSt2 pathways of amino acids, carbohydrates, glycans, lipids, vitamins, and nucleotide metabolism, which then directly affected the berry must (*R*² = −0.07; *p* = 0.020).

## 4 Discussion

### 4.1 Soil profiles did not identify clear causes of the decline

The pedological analysis of agricultural soils, also known as crop profiles, enables farmers to identify strategies for improving soil quality depending on the type of crop (Peigné et al., 2013). The objective here was to identify, on the one hand, any specific problems in the declining area that could explain the poor growth or even death of plants. Analysis of the soil profile in the S area did not distinguish any physical characteristics such as soil incident that could cause difficulty of rooting or possible water stress such as water retention. A few inconsequential brown spots were observed. Vine roots were present in the various deep horizons with variable sizes, both vertically and obliquely (**Supplementary Table S3**). Regarding chemical composition, overall, the observed values do not account for the very low vigor and yield of plants in the S area. The physicochemical values across the different horizons fell within the nutritional range established by Proffitt and Campbell-Clause (2012).

In both areas, all physicochemical parameter values decreased with depth, with the exception of CEC and alkaline phosphatase activity. The higher clay content in deep horizons compared to topsoil can explain the greater CEC found, which correlated with the higher amount of nutrient known to be available in their cationic form (Ca, Na, and K). Surprisingly, alkaline phosphatase activity was higher in deeper horizons, specifically in the S area, and was even significantly higher compared to the topsoil of the asymptomatic area. Phosphatase catalyzes the release of inorganic phosphorus from organic-bound phosphorus, which plays a crucial role in the P cycling. However, this activity was not correlated with P content, which was less than 2 mg g^−1^ below 25 cm deep. Tarafdar et al. (1989) reported an increase of acid phosphatase activity in deeper horizons (45–60 cm) of arid soil profiles in crop and tree lands and suggested that the root exudates were causing this increase of enzymatic activity in profound horizons. It has been suggested for acid phosphatase that stressed plants increase their activity under stress (Tadano and Sakai, 1991; Miller et al., 2001). In this case, symptomatic grapevines may increase the alkaline phosphatase enzymatic activity through root exudates as a “cry-for-help” strategy (Rolli et al., 2021).

Since the pits were dug at the end of the row close to the two S and AS areas, but not directly in the middle of these two areas, bulk soil samples from the inter-row were also analyzed. The observed values confirm those found in a previous sampling campaign in this plot (Darriaut et al., 2021). The slightly lower N concentration, consistent with a lower rate of nitrates in the soil of the S area compared to the AS area, is not reflected in the petiole nitrogen content. However, we can observe a concentration of assimilable nitrogen in the berries that is two times lower. Therefore, it seems more probable that this low concentration in the berries is more a consequence of the lower growth of the plants than a response to a low availability of N in the soil.

### 4.2 Microbial community structure was influenced by compartmentalization more than by soil status

Soil serves as the primary reservoir of the endophytic microbiota of grapevines (ZarraonaIndia et al., 2015; Marasco et al., 2018; Swift et al., 2021). Here, it is observed that 99.1% of bacterial OTUs and 94.4% of fungal OTUs of the root endosphere were also found in the rhizosphere, suggesting that less than 1% and 6% of root bacteria and fungi, respectively, are likely either obligate endophytes or endophytes transmitted by grafting or by the aerial parts of the plant. Across all compartments and soil conditions, the major bacterial phyla belonged to *Proteobacteria*, *Actinobacteriota*, *Acidobacteriota*, and *Firmicutes*, while the predominant fungal phyla were *Ascomycota* and *Basidiomycota*. These findings were consistent with previous works made in grapevine belowground compartments (Wei et al., 2018; Carbone et al., 2021; Marasco et al., 2022; Wang et al., 2023).

The structures of the fungal and bacterial communities from root endosphere were distinct from those found in soil. Consistent with previous research on grapevines, reduced bacterial and fungal richness as well as diversity were found in the root endosphere compared to soil samples (D’Amico et al., 2018; Marasco et al., 2018; Carbone et al., 2021; Swift et al., 2021; Darriaut et al., 2022a). The fungal root microbiome was influenced by soil status, revealing its sensitivity to soil composition. In the rhizosphere and bulk soil, α-diversity metrics were lower in the symptomatic condition compared to the asymptomatic condition, suggesting a dysregulation of the microbiome surrounding the declining grapevines. It has been proposed that higher diversity provided greater ecosystem processes, such as organic matter degradation (Maron et al., 2018). However, the α-diversity metrics of the bacterial and fungal root endosphere microbiome did not significantly differ between the conditions. It was not unexpected, given that endophytes often harbor more stable microbial communities compared to those found in the surrounding soil (Noman et al., 2021; Sun et al., 2021).

### 4.3 Latent fungi associated with grapevine diseases cohabited with potentially beneficial bacteria in the symptomatic area

These differences in microbiome structure might be linked to disease development, as a dysregulated root endosphere microbiome could facilitate pathogen colonization (Balbín-Suárez et al., 2021). Although symptoms of grapevine diseases were not observed in this plot, some fungal pathogens were detected in the different compartments, with higher abundance in symptomatic conditions. As reported in several studies, healthy plants may harbor potential latent endophytic fungal pathogens (Yang et al., 2018; Martínez-Diz et al., 2019; Manzotti et al., 2020), which can induce disease incidence under environmental constraints or during plant growth (Sosnowski et al., 2021). For instance, black foot and Petri diseases occur in vineyards supporting young grapevines (<5 years), whereas Esca complex and *Botryosphaeria* and *Eutypa* diebacks are usually observed in mature vineyards (Gramaje et al., 2018; Hrycan et al., 2020). In this specific case, the vineyard contained grapevines that were at least 10 years old, suggesting that the soil was the source of these detected fungi, which was supported by the higher abundance found in symptomatic samples. These findings were all in accordance with the previous results from bulk soil samples described in Darriaut et al. (2021) and Darriaut et al. (2023), confirming the microbial activity promotion and microbiome dysregulation in symptomatic soils. In an interesting way, *Clonostachys* was enriched in symptomatic rhizosphere, which is considered an efficient biocontrol agent for several grapevine trunk pathogens (Silva-Valderrama et al., 2021).

Additionally, using Limma-Voom differential analysis, it was found that *Legionella* was the most enriched bacterial taxa in both symptomatic rhizosphere and root endosphere, some species of which are known to cause Legionnaire’s disease in humans but no known pathological cases in grapevine (Casati et al., 2009). Another notable finding was the enriched *Methylosinus* in symptomatic rhizosphere, a diazotrophic methanotroph genus, known to be more abundant in soil and root environment with reduced nitrogen content (Ikeda et al., 2014), which is in line with our finding.

LEfSe analysis revealed enriched bacterial genera in symptomatic conditions belonging to *Rhizobium*, *Bradyrhizobium*, *Bacillus*, *Candidatus solibacter*, and *Niastella*. Through a metaproteome approach on the grapevine rhizosphere, Bona et al. (2019) demonstrated the involvement of several bacterial genera in protein and nitrogen metabolism, including *Bacillus* and *Bradyrhizobium*, here enriched in the symptomatic rhizosphere and root endosphere, respectively. The plant growth-promoting properties of *Rhizobium* and *Bradyrhizobium*, commonly found in in grapevine roots, are related to siderophore and phytohormone production, as well as nitrogen fixation and phosphate solubilization (Wright et al., 2022). The *Niastella* genus, enriched in symptomatic root samples, is known to produce indole acetic acid (IAA), which is the most common phytohormone with growth-promoting ability (Visioli et al., 2018). Interestingly, *C. solibacter* genus was predominant in soil contaminated by acid mine drainage or mudflat with long-term rice cultivation and was correlated with sulfur metabolism (Wang et al., 2018) and carbohydrate degradation (Zhang et al., 2019).

### 4.4 The functional diversity of the root and rhizosphere microbiomes is influenced by grapevine decline and affects the quality of the berry

Several microbe-recruiting compounds, including carbohydrates, amino acids, and organic acids, are transferred into the rhizosphere by roots through diffusion, ion channels, and vesicular transport (Vives-Peris et al., 2020). Eco-Plates measurements revealed greater degradation of amines, amino acids, carbohydrates, carboxylic acids, phenolic compounds, and polymers, in the symptomatic rhizosphere and bulk soils compared to asymptomatic samples. A decrease in these physiological profiles of microbial communities in soils with lower organic matter content was reported in a multisite study (Rutgers et al., 2016), which is not consistent with our findings, as no significant difference in organic matter was found between S and AS areas in the topsoil.

Eco-Plates do not provide in-depth analysis, and further investigation would be required to identify the taxa responsible for this high microbial activity in the symptomatic condition, especially in the rhizosphere compartment, which displayed increased degradation of substrates. In parallel, the metabolic functions of bacterial communities were predicted using PICRUSt2, which has been widely used to explain bacterial functioning diversity in plants, but remains rare for grapevine studies (ZarraonaIndia et al., 2015; Rosado et al., 2022). In this specific case, carbohydrates and amino acid pathways were upregulated in asymptomatic rhizosphere samples using PICRUSt2. In addition, higher functional diversity with lower microbial richness and reduced fungal diversity in symptomatic bulk soil were reported. These findings suggest a metabolic redundancy in the symptomatic soils, since functions like the biogeochemical processes in the vineyard might be related to distinct taxonomic composition but with similar metabolic functions (Griggs et al., 2021). While few studies have compared the functional inference and metabolic processes, the predicted functions of roots in this case, using PICRUSt2, were concordant with some Eco-Plates measurements from rhizosphere compartment, such as the upregulation of the metabolism of carbohydrates and amino acid pathways. This finding has led to the hypothesis that the high capacity to degrade carbohydrates and amino acids in the symptomatic rhizosphere is linked to the proximal bacterial endophytic communities. Zhao et al. (2022) reported that some predicted microbial functionalities were correlated to metabolic processes assessed with Biolog™ technology, particularly those related to amino acids and carbohydrates. Furthermore, some fungal and bacterial genera, such as *Mycobacterium*, *Niastella*, and *Dictyochaeta*, were negatively correlated to these assessed metabolic processes, which are even considered as microorganisms involved in numerous carbon- and nitrogen-based degradation (Bill et al., 2021; Mauran et al., 2021). However, the need for culture-dependent approaches to validate the effects of specific microbial taxa and functions on vine fitness should be further developed, as suggested by Fournier et al. (2022).

According to the PLS-PM, soil status and microbial root functionality based on various metabolism pathways (e.g., carbohydrates and amino acids) potentially affected the composition and the quality of the berry must in a synergistic manner. Although these findings are only assumptions, the contribution of the roots and soil microbiome to the berry composition should be further explored, since very few studies have investigated these interactions (Darriaut et al., 2022b). Since wine quality is linked to the quality of berries, the must composition is one of the most concerning components for the winegrowers. Must composition is known to correlate with water deficit and soil composition (Brillante et al., 2018), and its microbiota, mainly composed of yeast, has been studied for its effects on the wine making process (Martins et al., 2012; Morgan et al., 2017). Sugars, nitrogen content (assimilable N, NOPA, and ammoniacal), and acidity in must composition are considered as quality indicators of grapes (Downey et al., 2006; Cagnasso et al., 2008). In addition, inadequate assimilable nitrogen content in must composition may cause problems in yeast functioning and, therefore, wine fermentation, which can be alleviated by adding nitrogen (Paolini et al., 2016). Here, we found less malic and tartaric acids in symptomatic samples compared to asymptomatic ones, showing a potential incidence in wine quality. Similarly, assimilable N and NOPA, which are essential compounds in yeast nutrition and therefore of primary importance in must fermentation (Vilanova et al., 2007), were lower in the symptomatic area but higher than previously reported nitrogen deficiencies values of 140 mg N/L (Bely et al., 1990).

This study revealed microbiome dysbiosis in the root endosphere, rhizosphere, and bulk soils of a vineyard experiencing unexplained decline. Transplanting bulk soil or even rhizosphere microbiome from asymptomatic areas to symptomatic ones would be an interesting process to set up in order to explore whether this dysbiosis might be alleviated. This type of experiment was already investigated in tomato experiencing wilt disease, highlighting the importance of the rhizosphere microbiome toward the host fitness (Kwak et al., 2018; Zhou et al., 2023). Similarly, transplanting healthy soil microbiome into a symptomatic environment would be another way to assess the belowground microbiome ability to influence the berry composition. Characteristics of the deep soil horizons could not explain the declining symptoms affiliated with low vigor and yield and poor quality of the berries. Symptomatic samples showed enriched taxa of potentially beneficial bacteria that might be explained by the presence of latent fungal genera associated with grapevine diseases. Conversely, the frequency of mycorrhization with arbuscular mycorrhizal fungi and the abundance in *Rhizophagus* sequences were higher in roots of vines grown in asymptomatic areas. The berry composition appeared to be synergistically linked to the functional diversity in roots and to the symptomatic trait of the area, highlighting the crucial role of root microbiome. To gain a comprehensive overview of stressed vineyards, holistic approaches including microbiome taxonomic and functional diversity should be explored. This diagnosis could facilitate the implementation of cultural strategies, such as the addition of bacterial and fungal biocontrol consortia or biostimulants, in order to correct this dysbiosis.

## Data availability statement

The datasets presented in this study can be found in online repositories. The names of the repository/repositories and accession number(s) can be found below: https://www.ncbi.nlm.nih.gov/bioproject/PRJNA868524.

## Author contributions

RD: Investigation, Methodology, Data curation, Formal analysis, Visualization, Writing – original draft, Writing – review & editing. TM: Investigation, Writing – review & editing. VLai: Investigation, Writing – review & editing. JT: Data curation, Formal analysis, Methodology, Writing – review & editing. EM: Conceptualization, Investigation, Writing – review & editing. IMP: Conceptualization, Funding acquisition, Writing – review & editing. GM: Investigation, Writing – review & editing. Vlau: Conceptualization, Funding acquisition, Project administration, Supervision, Writing – original draft, Writing – review & editing.

## References

[B1] Andrews S. (2010) FastQC: a quality control tool for high throughput sequence data. Available online at: http://www.bioinformatics.babraham.ac.uk/projects/fastqc.

[B2] Arnault G. Mony C. Vandenkoornhuyse P. (2023). Plant microbiota dysbiosis and the Anna Karenina Principle. Trends Plant Sci. 28, 18–30. doi: 10.1016/j.tplants.2022.08.012 36127241

[B3] Balbín-Suárez A. Jacquiod S. Rohr A.-D. Liu B. Flachowsky H. Winkelmann T.. (2021). Root exposure to apple replant disease soil triggers local defense response and rhizoplane microbiome dysbiosis. FEMS Microbiol. Ecol. 97, fiab031. doi: 10.1093/femsec/fiab031 33587112

[B4] Banerjee S. Schlaeppi K. and van der Heijden M. G. A. (2018). Keystone taxa as drivers of microbiome structure and functioning. Nat. Rev. Microbiol. 16, 567–576. doi: 10.1038/s41579-018-0024-1 29789680

[B5] Banerjee S. Walder F. Büchi L. Meyer M. Held A. Y. Gattinger A.. (2019). Agricultural intensification reduces microbial network complexity and the abundance of keystone taxa in roots. ISME J. 13, 1722–1736. doi: 10.1038/s41396-019-0383-2 30850707 PMC6591126

[B6] Bekris F. Vasileiadis S. Papadopoulou E. Samaras A. Testempasis S. Gkizi D.. (2021). Grapevine wood microbiome analysis identifies key fungal pathogens and potential interactions with the bacterial community implicated in grapevine trunk disease appearance. Environ. microbio. 16, 1–17. doi: 10.1186/s40793-021-00390-1 PMC8642934 34863281

[B7] Bely M. Sablayrolles J.-M. Barre P. (1990). Automatic detection of assimilable nitrogen deficiencies during alcoholic fermentation in oenological conditions. J. Ferment. Bioeng. 70, 246–252. doi: 10.1016/0922-338X(90)90057-4

[B8] Bettenfeld P. Cadena i Canals J. Jacquens L. Fernandez O. Fontaine F. van Schaik E.. (2022). The microbiota of the grapevine holobiont: A key component of plant health. J. Adv. Res. 40, 1–15. doi: 10.1016/j.jare.2021.12.008 36100319 PMC9481934

[B9] Bill M. Chidamba L. Gokul J. K. Labuschagne N. Korsten L. (2021). Bacterial community dynamics and functional profiling of soils from conventional and organic cropping systems. Appl. Soil Ecol. 157, 103734. doi: 10.1016/j.apsoil.2020.103734

[B10] Bona E. Massa N. Novello G. Boatti L. Cesaro P. Todeschini V.. (2019). Metaproteomic characterization of Vitis vinifera rhizosphere. FEMS Microbiol. Ecol. 95, fiy204. doi: 10.1093/femsec/fiy204 30307579

[B11] Brillante L. Mathieu O. Lévêque J. van Leeuwen C. Bois B. (2018). Water status and must composition in grapevine cv. Chardonnay with different soils and topography and a mini meta-analysis of the δ 13 C/water potentials correlation. J. Sci. Food Agric. 98, 691–697. doi: 10.1002/jsfa.8516 28671281

[B12] Cagnasso E. Rolle L. Caudana A. Gerbi V. (2008). Relationship between grape phenolic maturity and red wine phenolic composition. Ital. J. Food Sci. 20, 365–380.

[B13] Carbone M. J. Alaniz S. Mondino P. Gelabert M. Eichmeier A. Tekielska D.. (2021). Drought influences fungal community dynamics in the grapevine rhizosphere and root microbiome. J. Fungi 7, 686. doi: 10.3390/jof7090686 PMC8468433 34575724

[B14] Casati S. Gioria-Martinoni A. Gaia V. (2009). Commercial potting soils as an alternative infection source of Legionella pneumophila and other Legionella species in Switzerland. Clin. Microbiol. Infect. 15, 571–575. doi: 10.1111/j.1469-0691.2009.02742.x 19392903

[B15] Chakraborty S. Luck J. Hollaway G. Freeman A. Norton R. Garrett K. A.. (2008). Impacts of global change on diseases of agricultural crops and forest trees. CAB Rev. Perspect. Agric. Vet. Sci. Nutr. Nat. Resour. 3, 1–15. doi: 10.1079/PAVSNNR20083054

[B16] Chen Q.-L. Ding J. Zhu Y.-G. He J.-Z. Hu H.-W. (2020). Soil bacterial taxonomic diversity is critical to maintaining the plant productivity. Environ. Int. 140, 105766. doi: 10.1016/j.envint.2020.105766 32371308

[B17] Chuche J. Danet J.-L. Rivoal J.-B. Arricau-Bouvery N. Thiéry D. (2018). Minor cultures as hosts for vectors of extensive crop diseases: Does Salvia sclarea act as a pathogen and vector reservoir for lavender decline? J. Pest Sci. . 91, 145–155. doi: 10.1007/s10340-017-0885-5

[B18] Cienciala E. Tumajer J. Zatloukal V. Beranová J. Holá Š. Hůnová I.. (2017). Recent spruce decline with biotic pathogen infestation as a result of interacting climate, deposition and soil variables. Eur. J. For. Res. 136, 307–317. doi: 10.1007/s10342-017-1032-9

[B19] Cobos R. Ibañez A. Diez-Galán A. Calvo-Peña C. Ghoreshizadeh S. Coque J. J. R. (2022). The grapevine microbiome to the rescue: implications for the biocontrol of trunk diseases. Plants 11, 840. doi: 10.3390/plants11070840 35406820 PMC9003034

[B20] Cox C. M. Garrett K. A. Bockus W. W. (2005). Meeting the challenge of disease management in perennial grain cropping systems. Renew. Agric. Food Syst. 20, 15–24. doi: 10.1079/RAF200495

[B21] D’Amico F. Candela M. Turroni S. Biagi E. Brigidi P. Bega A.. (2018). The rootstock regulates microbiome diversity in root and rhizosphere compartments of vitis vinifera cultivar lambrusco. Front. Microbiol. 9. doi: 10.3389/fmicb.2018.02240 PMC6169447 30319569

[B22] Darriaut R. Antonielli L. Martins G. Ballestra P. Vivin P. Marguerit E.. (2022a). Soil composition and rootstock genotype drive the root associated microbial communities in young grapevines. Front. Microbiol. 13. doi: 10.3389/fmicb.2022.1031064 PMC9685171 36439844

[B23] Darriaut R. Lailheugue V. Masneuf-Pomarède I. Marguerit E. Martins G. Compant S.. (2022b). Grapevine rootstock and soil microbiome interactions: Keys for a resilient viticulture. Hortic. Res. 9, uhac019. doi: 10.1093/hr/uhac019 35184168 PMC8985100

[B24] Darriaut R. Martins G. Dewasme C. Mary S. Darrieutort G. Ballestra P.. (2021). Grapevine decline is associated with difference in soil microbial composition and activity. OENO One 55, 67–84. doi: 10.20870/oeno-one.2021.55.3.4626

[B25] Darriaut R. Tran J. Martins G. Ollat N. Masneuf-Pomarède I. Lauvergeat V. (2023). In grapevine decline, microbiomes are affected differently in symptomatic and asymptomatic soils. Appl. Soil Ecol. 183, 104767. doi: 10.1016/j.apsoil.2022.104767

[B26] Destrac A. Flutre T. Renaud C. Morin E. Durand L. Delrot S.. (2015). The use of Fourier transform infrared spectroscopy in phenotyping berries from the grapevine Vitis Vinifera L. @ in 19. Journées Internationales Viticult. GiESCO 810, 641–645.

[B27] Deyett E. Rolshausen P. E. (2019). Temporal dynamics of the sap microbiome of grapevine under high Pierce’s disease pressure. Front. Plant Sci. 10, 641–645. doi: 10.3389/fpls.2019.01246 31681363 PMC6805966

[B28] Douglas G. M. Maffei V. J. Zaneveld J. R. Yurgel S. N. Brown J. R. Taylor C. M.. (2020). PICRUSt2 for prediction of metagenome functions. Nat. Biotechnol. 38, 685–688. doi: 10.1038/s41587-020-0548-6 32483366 PMC7365738

[B29] Downey M. O. Dokoozlian N. K. Krstic M. P. (2006). Cultural practice and environmental impacts on the flavonoid composition of grapes and wine: A review of recent research. Am. J. Enol. Vitic. 57, 257–268. doi: 10.5344/ajev.2006.57.3.257

[B30] Edgar R. C. Haas B. J. Clemente J. C. Quince C. Knight R. (2011). UCHIME improves sensitivity and speed of chimera detection. Bioinformatics 27, 2194–2200. doi: 10.1093/bioinformatics/btr381 21700674 PMC3150044

[B31] Escudié F. Auer L. Bernard M. Mariadassou M. Cauquil L. Vidal K.. (2018). FROGS: find, rapidly, OTUs with galaxy solution. Bioinformatics 34, 1287–1294. doi: 10.1093/bioinformatics/btx791 29228191

[B32] Fierer N. (2017). Embracing the unknown: disentangling the complexities of the soil microbiome. Nat. Rev. Microbiol. 15, 579–590. doi: 10.1038/nrmicro.2017.87 28824177

[B33] Fournier P. Pellan L. Barroso-Bergadà D. Bohan D. A. Candresse T. Delmotte F.. (2022). “The functional microbiome of grapevine throughout plant evolutionary history and lifetime,” in Advances in Ecological Research (United States: Academic Press), 27–99. doi: 10.1016/bs.aecr.2022.09.001

[B34] Fraser T. Brown P. D. (2017). Temperature and oxidative stress as triggers for virulence gene expression in pathogenic Leptospira spp. Front. Microbiol. 8. doi: 10.3389/fmicb.2017.00783 PMC5423269 28536558

[B35] Ginnan N. A. Dang T. Bodaghi S. Ruegger P. M. McCollum G. England G.. (2020). Disease-induced microbial shifts in citrus indicate microbiome-derived responses to huanglongbing across the disease severity spectrum. Phytobio. J. 4, 375–387. doi: 10.1094/PBIOMES-04-20-0027-R

[B36] Gramaje D. Úrbez-Torres J. R. Sosnowski M. R. (2018). Managing grapevine trunk diseases with respect to etiology and epidemiology: current strategies and future prospects. Plant Dis. 102, 12–39. doi: 10.1094/PDIS-04-17-0512-FE 30673457

[B37] Griggs R. G. Steenwerth K. L. Mills D. A. Cantu D. Bokulich N. A. (2021). Sources and assembly of microbial communities in vineyards as a functional component of winegrowing. Front. Microbiol. 12. doi: 10.3389/fmicb.2021.673810 PMC8076609 33927711

[B38] Hrycan J. Hart M. Bowen P. Forge T. Úrbez-Torres J. R. (2020). Grapevine trunk disease fungi: their roles as latent pathogens and stress factors that favour disease development and symptom expression. Phytopathol. Mediterr. 59, 395–424. doi: 10.14601/Phyto-11275

[B39] Ikeda S. Sasaki K. Okubo T. Yamashita A. Terasawa K. Bao Z.. (2014). Low nitrogen fertilization adapts rice root microbiome to low nutrient environment by changing biogeochemical functions. Microbes Environ. 29, 50–59. doi: 10.1264/jsme2.ME13110 24463575 PMC4041235

[B40] Karimi B. Dequiedt S. Terrat S. Jolivet C. Arrouays D. Wincker P.. (2019). Biogeography of soil bacterial networks along a gradient of cropping intensity. Sci. Rep. 9, 3812. doi: 10.1038/s41598-019-40422-y 30846759 PMC6405751

[B41] Karimi B. Villerd J. Dequiedt S. Terrat S. Chemidlin-Prévost Bouré N. Djemiel C.. (2020). Biogeography of soil microbial habitats across France. Glob. Ecol. Biogeogr. 29, 1399–1411. doi: 10.1111/geb.13118

[B42] Köberl M. Dita M. Martinuz A. Staver C. Berg G. (2017). Members of Gammaproteobacteria as indicator species of healthy banana plants on Fusarium wilt-infested fields in Central America. Sci. Rep. 7, 45318. doi: 10.1038/srep45318 28345666 PMC5366900

[B43] Kwak M. J. Kong H. G. Choi K. Kwon S. K. Song J. Y. Lee J.. (2018). Rhizosphere microbiome structure alters to enable wilt resistance in tomato. Nat. Biotechnol. 36, 1100–1109. doi: 10.1038/nbt.4232 30295674

[B44] Lee S. M. Kong H. G. Song G. C. Ryu C. M. (2021). Disruption of Firmicutes and Actinobacteria abundance in tomato rhizosphere causes the incidence of bacterial wilt disease. ISME J. 15, 330–347. doi: 10.1038/s41396-020-00785-x 33028974 PMC7852523

[B45] Lin Y. Ye G. Kuzyakov Y. Liu D. Fan J. Ding W. (2019). Long-term manure application increases soil organic matter and aggregation, and alters microbial community structure and keystone taxa. Soil Biol. Biochem. 134, 187–196. doi: 10.1016/j.soilbio.2019.03.030

[B46] Liu D. Howell K. (2021). Community succession of the grapevine fungal microbiome in the annual growth cycle. Environ. Microbiol 23, 1842–1857. doi: 10.1111/1462-2920.15172 32686214

[B47] Mahé F. Rognes T. Quince C. de Vargas C. Dunthorn M. (2014). Swarm: robust and fast clustering method for amplicon-based studies. PeerJ 2, e593. doi: 10.7717/peerj.593 25276506 PMC4178461

[B48] Manzotti A. Bergna A. Burow M. Jørgensen H. J. L. Cernava T. Berg G.. (2020). Insights into the community structure and lifestyle of the fungal root endophytes of tomato by combining amplicon sequencing and isolation approaches with phytohormone profiling. FEMS Microbiol. Ecol. 96, fiaa052. doi: 10.1093/femsec/fiaa052 32239208 PMC7174037

[B49] Marasco R. AlTurkey H. Fusi M. Brandi M. Ghiglieno I. Valenti L.. (2022). Rootstock–scion combination contributes to shape diversity and composition of microbial communities associated with grapevine root system. Environ. Microbiol. 24, 3791–3808. doi: 10.1111/1462-2920.16042 35581159 PMC9544687

[B50] Marasco R. Rolli E. Fusi M. Michoud G. Daffonchio D. (2018). Grapevine rootstocks shape underground bacterial microbiome and networking but not potential functionality. Microbiome 6, 3. doi: 10.1186/s40168-017-0391-2 29298729 PMC5751889

[B51] Marçais B. Bréda N. (2006). Role of an opportunistic pathogen in the decline of stressed oak trees. J. Ecol. 94, 1214–1223. doi: 10.1111/j.1365-2745.2006.01173.x

[B52] Maron P.-A. Sarr A. Kaisermann A. Lévêque J. Mathieu O. Guigue J.. (2018). High microbial diversity promotes soil ecosystem functioning. Appl. Environ. Microbiol. 84, e02738–e02717. doi: 10.1128/AEM.02738-17 29453268 PMC5930326

[B53] Martin M. (2011). Cutadapt removes adapter sequences from high-throughput sequencing reads. EMBnet. journal 17, 10–12. doi: 10.14806/ej.17.1.200

[B54] Martínez-Diz M. del P. Andrés-Sodupe M. Bujanda R. Díaz-Losada E. Eichmeier A.. (2019). Soil-plant compartments affect fungal microbiome diversity and composition in grapevine. Fungal Ecol. 41, 234–244. doi: 10.1016/j.funeco.2019.07.003

[B55] Martins G. Miot-Sertier C. Lauga B. Claisse O. Lonvaud-Funel A. Soulas G.. (2012). Grape berry bacterial microbiota: Impact of the ripening process and the farming system. Int. J. Food Microbiol. 158, 93–100. doi: 10.1016/j.ijfoodmicro.2012.06.013 22809638

[B56] Mauran S. Perera N. T. Perera I. C. (2021). MxyR of mycobacterium tuberculosis responds to xylan; an unusual ligand for a marR family transcriptional regulator. Mol. Biol. 55, 870–883. doi: 10.1134/S0026893321050162 35082266

[B57] Miller S. S. Liu J. Allan D. L. Menzhuber C. J. Fedorova M. Vance C. P. (2001). Molecular control of acid phosphatase secretion into the rhizosphere of proteoid roots from phosphorus-stressed white lupin. Plant Physiol. 127, 594–606. doi: 10.1104/pp.010097 11598233 PMC125094

[B58] Morgan H. H. du Toit M. Setati M. E. (2017). The grapevine and wine microbiome: insights from high-throughput amplicon sequencing. Front. Microbiol. 8. doi: 10.3389/fmicb.2017.00820 PMC5425579 28553266

[B59] Nanetti E. Palladino G. Scicchitano D. Trapella G. Cinti N. Fabbrini M.. (2023). Composition and biodiversity of soil and root-associated microbiome in Vitis vinifera cultivar Lambrusco distinguish the microbial terroir of the Lambrusco DOC protected designation of origin area on a local scale. Front. Microbiol. 14. doi: 10.3389/fmicb.2023.1108036 PMC9992870 36910169

[B60] Nerva L. Zanzotto A. Gardiman M. Gaiotti F. Chitarra W. (2019). Soil microbiome analysis in an ESCA diseased vineyard. Soil Biol. Biochem. 135, 60–70. doi: 10.1016/j.soilbio.2019.04.014

[B61] Noman M. Ahmed T. Ijaz U. Shahid M. Azizullah Li D.. (2021). Plant–Microbiome crosstalk: Dawning from composition and assembly of microbial community to improvement of disease resilience in plants. Int. J. Mol. Sci. 22, 6852. doi: 10.3390/ijms22136852 34202205 PMC8269294

[B62] Pacifico D. Squartini A. Crucitti D. Barizza E. Lo Schiavo F. Muresu R.. (2019). The role of the endophytic microbiome in the grapevine response to environmental triggers. Front. Plant Sci. 10. doi: 10.3389/fpls.2019.01256 PMC6794716 31649712

[B63] Pandey P. Senthil-Kumar M. (2019). Plant-pathogen interaction in the presence of abiotic stress: What do we know about plant responses? Plant Physiol. Rep. 24, 541–549. doi: 10.1007/s40502-019-00483-7

[B64] Paolini M. Ziller L. Bertoldi D. Bontempo L. Larcher R. Nicolini G.. (2016). δ15N from soil to wine in bulk samples and proline. J. Mass Spectrom. 51, 668–674. doi: 10.1002/jms.3824 27479606

[B65] Peigné J. Vian J.-F. Cannavacciuolo M. Lefevre V. Gautronneau Y. Boizard H. (2013). Assessment of soil structure in the transition layer between topsoil and subsoil using the profil cultural method. Soil Tillage Res. 127, 13–25. doi: 10.1016/j.still.2012.05.014

[B66] Phillips J. M. Hayman D. S. (1970). Improved procedures for clearing roots and staining parasitic and vesicular-arbuscular mycorrhizal fungi for rapid assessment of infection. Trans. Br. Mycol. Soc 55, 158–IN18. doi: 10.1016/S0007-1536(70)80110-3

[B67] Proffitt T. Campbell-Clause J. (2012). Managing grapevine nutrition and vineyard soil health (Claremont: Wines of Western Australia). Available at: http://www.winewa.asn.au/.

[B68] Rognes T. Flouri T. Nichols B. Quince C. Mahé F. (2016). VSEARCH: a versatile open source tool for metagenomics. PeerJ 4, e2584. doi: 10.7717/peerj.2584 27781170 PMC5075697

[B69] Rolli E. Vergani L. Ghitti E. Patania G. Mapelli F. Borin S. (2021). ‘Cry-for-help’ in contaminated soil: a dialogue among plants and soil microbiome to survive in hostile conditions. Environ. Microbiol. 23, 5690–5703. doi: 10.1111/1462-2920.15647 34139059 PMC8596516

[B70] Rosado D. Lores M. Ramos-Tapia I. Crandall K. A. Pérez-Losada M. Domínguez J. (2022). Integrated fertilization with bagasse vermicompost changes the microbiome of mencía must and wine. Fermentation 8, 357. doi: 10.3390/fermentation8080357

[B71] Rutgers M. Wouterse M. Drost S. M. Breure A. M. Mulder C. Stone D.. (2016). Monitoring soil bacteria with community-level physiological profiles using BiologTM ECO-plates in the Netherlands and Europe. Appl. Soil Ecol. 97, 23–35. doi: 10.1016/j.apsoil.2015.06.007

[B72] Silva-Valderrama I. Toapanta D. de los Angeles Miccono M. Lolas M. Díaz G. A. Cantu D.. (2021). Biocontrol potential of grapevine endophytic and rhizospheric fungi against trunk pathogens. Front. Microbiol. 11. doi: 10.3389/fmicb.2020.614620 PMC7817659 33488557

[B73] Sosnowski M. R. Ayres M. R. Scott E. S. (2021). The Influence of Water Deficit Stress on the Grapevine Trunk Disease Pathogens Eutypa lata and Diplodia seriata. Plant Dis. 105, 2217–2221. doi: 10.1094/PDIS-07-20-1538-RE 33141641

[B74] Sun A. Jiao X. Y. Chen Q. Wu A. L. Zheng Y. Lin Y. X.. (2021). Microbial communities in crop phyllosphere and root endosphere are more resistant than soil microbiota to fertilization. Soil Biol. Biochem. 153, 108113. doi: 10.1016/j.soilbio.2020.108113

[B75] Suter B. Destrac Irvine A. Gowdy M. Dai Z. van Leeuwen C. (2021). Adapting wine grape ripening to global change requires a multi-trait approach. Front. Plant Sci. 12. doi: 10.3389/fpls.2021.624867 PMC7893094 33613606

[B76] Swift J. F. Hall M. E. Harris Z. N. Kwasniewski M. T. Miller A. J. (2021). Grapevine microbiota reflect diversity among compartments and complex interactions within and among root and shoot systems. Microorganisms 9, 92. doi: 10.3390/microorganisms9010092 33401756 PMC7823683

[B77] Tadano T. Sakai H. (1991). Secretion of acid phosphatase by the roots of several crop species under phosphorus-deficient conditions. Soil Sci. Plant Nutr. 37, 129–140. doi: 10.1080/00380768.1991.10415018

[B78] Tarafdar J. C. Kiran B. Rao A. V. (1989). Phosphatase activity and distribution of phosphorus in arid soil profiles under different land use patterns. J. Arid Environ. 16, 29–34. doi: 10.1016/S0140-1963(18)31044-9

[B79] Trivedi P. Leach J. E. Tringe S. G. Sa T. Singh B. K. (2020). Plant–microbiome interactions: from community assembly to plant health. Nat. Rev. Microbiol. 18, 607–621. doi: 10.1038/s41579-020-0412-1 32788714

[B80] Trouvelot A. Kough J. L. Gianinazzi-Pearson V. (1986). Mesure du taux de mycorhization VA d’un systeme radiculaire. Recherche de methodes d’estimation ayant une signification fonctionnelle. In: Gianinazzi V. Eds,. Physiological and Genetical Aspects of Mycorrhizae, INRA, Paris, 217–221.

[B81] Van Leeuwen C. Trégoat O. Choné X. Bois B. Pernet D. Gaudillère J.-P. (2009). Vine water status is a key factor in grape ripening and vintage quality for red Bordeaux wine. How can it be assessed for vineyard management purposes? OENO One 43, 121–134. doi: 10.20870/oeno-one.2009.43.3.798

[B82] Vilanova M. Ugliano M. Varela C. Siebert T. Pretorius I. S. Henschke P. A. (2007). Assimilable nitrogen utilisation and production of volatile and non-volatile compounds in chemically defined medium by Saccharomyces cerevisiae wine yeasts. Appl. Microbiol. Biotechnol. 77, 145–157. doi: 10.1007/s00253-007-1145-z 17846763

[B83] Visioli G. Sanangelantoni A. M. Vamerali T. Dal Cortivo C. Blandino M. (2018). 16S rDNA profiling to reveal the influence of seed-applied biostimulants on the rhizosphere of young maize plants. Molecules 23, 1461. doi: 10.3390/molecules23061461 29914131 PMC6100521

[B84] Vives-Peris V. de Ollas C. Gómez-Cadenas A. Pérez-Clemente R. M. (2020). Root exudates: from plant to rhizosphere and beyond. Plant Cell Rep. 39, 3–17. doi: 10.1007/s00299-019-02447-5 31346716

[B85] Wang B. Wang X. Wang Z. Zhu K. Wu W. (2023). Comparative metagenomic analysis reveals rhizosphere microbial community composition and functions help protect grapevines against salt stress. Front. Microbiol. 14. doi: 10.3389/fmicb.2023.1102547 PMC9987714 36891384

[B86] Wang H. Zeng Y. Guo C. Bao Y. Lu G. Reinfelder J. R.. (2018). Bacterial, archaeal, and fungal community responses to acid mine drainage-laden pollution in a rice paddy soil ecosystem. Sci. Total Environ. 616–617, 107–116. doi: 10.1016/j.scitotenv.2017.10.224 29107775

[B87] Wei Z. Gu Y. Friman V.-P. Kowalchuk G. A. Xu Y. Shen Q.. (2019). Initial soil microbiome composition and functioning predetermine future plant health. Sci. Adv. 5, eaaw0759. doi: 10.1126/sciadv.aaw0759 31579818 PMC6760924

[B88] Wei Y. Wu Y. Yan Y. Zou W. Xue J. Ma W.. (2018). High-throughput sequencing of microbial community diversity in soil, grapes, leaves, grape juice and wine of grapevine from China. PloS One 13, e0193097. doi: 10.1371/journal.pone.0193097 29565999 PMC5863948

[B89] Wright A. H. Ali S. Migicovsky Z. Douglas G. M. Yurgel S. Bunbury-Blanchette A.. (2022). A characterization of a cool-climate organic vineyard’s microbiome. Phytobio. J. 6, 69–82. doi: 10.1094/PBIOMES-03-21-0019-R

[B90] Yang H. Ye W. Ma J. Zeng D. Rong Z. Xu M.. (2018). Endophytic fungal communities associated with field-grown soybean roots and seeds in the Huang-Huai region of China. PeerJ 6, e4713. doi: 10.7717/peerj.4713 29736345 PMC5933319

[B91] ZarraonaIndia I. Owens S. M. Weisenhorn P. West K. Hampton-Marcell J. Lax S.. (2015). The soil microbiome influences grapevine-associated microbiota. MBio 6, e02527–e02514. doi: 10.1128/mBio.02527-14 25805735 PMC4453523

[B92] Zhang Y. Li Q. Chen Y. Dai Q. Hu J. (2019). Dynamic Change in Enzyme Activity and Bacterial Community with long-term rice Cultivation in Mudflats. Curr. Microbiol. 76, 361–369. doi: 10.1007/s00284-019-01636-5 30684025

[B93] Zhao Q. Xie F. Zhang F. Zhou K. Sun H. Zhao Y.. (2022). Analysis of bacterial community functional diversity in late-stage shrimp (Litopenaeus vannamei) ponds using Biolog EcoPlates and PICRUSt2. Aquaculture 546, 737288. doi: 10.1016/j.aquaculture.2021.737288

[B94] Zhou X. Zhang J. u Rahman M. K. Gao D. Wei Z. Wu F.. (2023). Interspecific plant interaction via root exudates structures the disease suppressiveness of rhizosphere microbiomes. Mol. Plant 16, 849–864. doi: 10.1016/j.molp.2023.03.009 36935607

