# Supplementary material for: Microbial dysbiosis in roots and rhizosphere of grapevines experiencing decline is associated with active metabolic functions
Source: Front Plant Sci. 2024 Apr 2;15:1358213. doi: 10.3389/fpls.2024.1358213 (PMC11018932; doi:10.3389/fpls.2024.1358213)
Supplement: Supplementary file 1 [file DataSheet_1.pdf]

Supplementary Material

Stressed vines associated with soil and root microbiome dysbiosis display potentially active metabolic pathways

Romain Darriaut[1a], Tania Marzari[1b], Vincent Lailheugue[1], Joseph Tran[1], Guilherme Martins[2,3], Elisa Marguerit[1], Isabelle Masneuf-Pomarède[2,3], Virginie Lauvergeat[1*]
[1] EGFV, Univ. Bordeaux, Bordeaux Sciences Agro, INRAE, ISVV, F-33882, Villenave d'Ornon, France
[2] Université de Bordeaux, INRAE, Bordeaux INP, UR Œnologie EA 4577, USC 1366, ISVV, 33140 Villenave d'Ornon, France
[3] Bordeaux Sciences Agro, 1 cours du Général de Gaulle, 33170 Gradignan, France
[a] Present address: Univ Rennes, CNRS, ECOBIO (Ecosystèmes, biodiversité, évolution) - UMR 6553, F-35000 Rennes, France
[b] Present address: Agroécologie, CNRS, INRAE, Institut Agro, Univ. Bourgogne, Univ. Bourgogne Franche-Comté, Dijon, France

*** Correspondence:**
Virginie Lauvergeat
virginie.lauvergeat@inrae.fr

1 Supplementary Figures

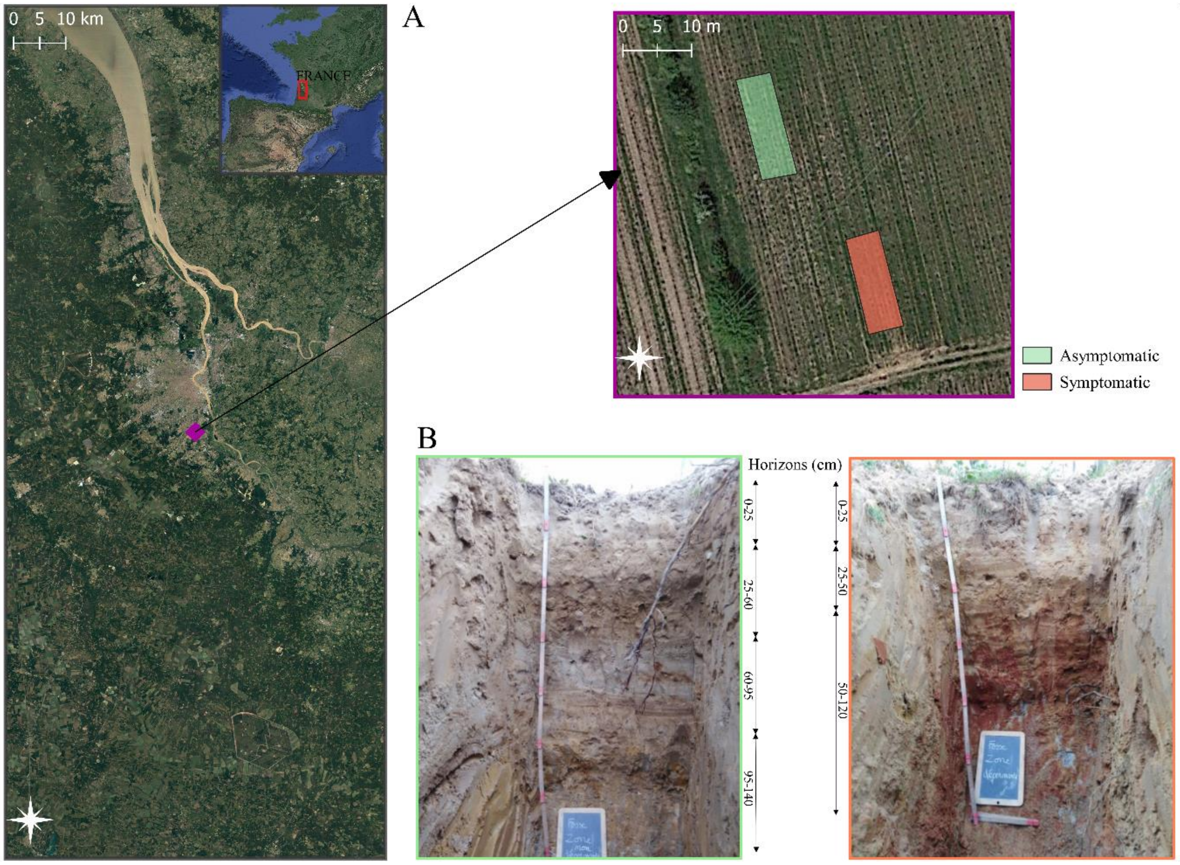

Supplementary Figure S1. Geographical location of studied vineyard in Bordeaux region, from Graves terroir (GPS coordinates: 44°45'13.0"N 0°33'24.8"W). **(A)** Areas of sampled asymptomatic (AS, green) and symptomatic (S, orange) vines. **(B)** Soil profiles generated in AS (left) and S (right) zones across the different horizons.

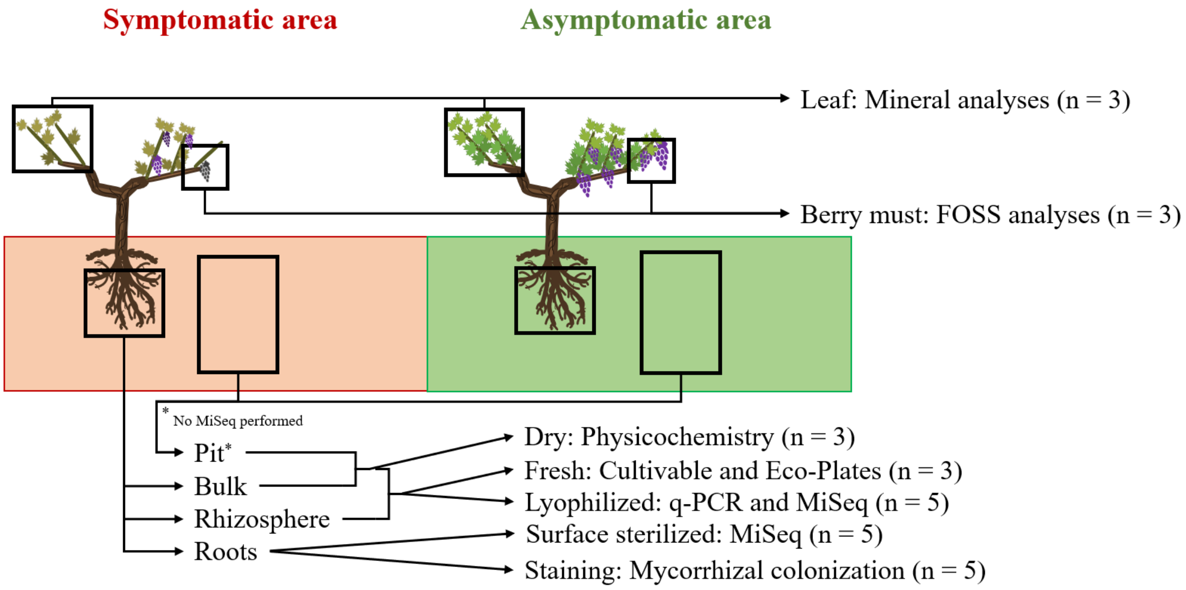

Supplementary Figure S2. Schematic overview of the samplings and measurements made on leaves, berries, soil from the pit, bulk, rhizosphere, and root compartments. No MiSeq analysis was done for pit samples. For each analysis, the number of biological replicates was indicated under brackets (n). Bulk soil, rhizosphere and root samplings were done on five selected vines per area.

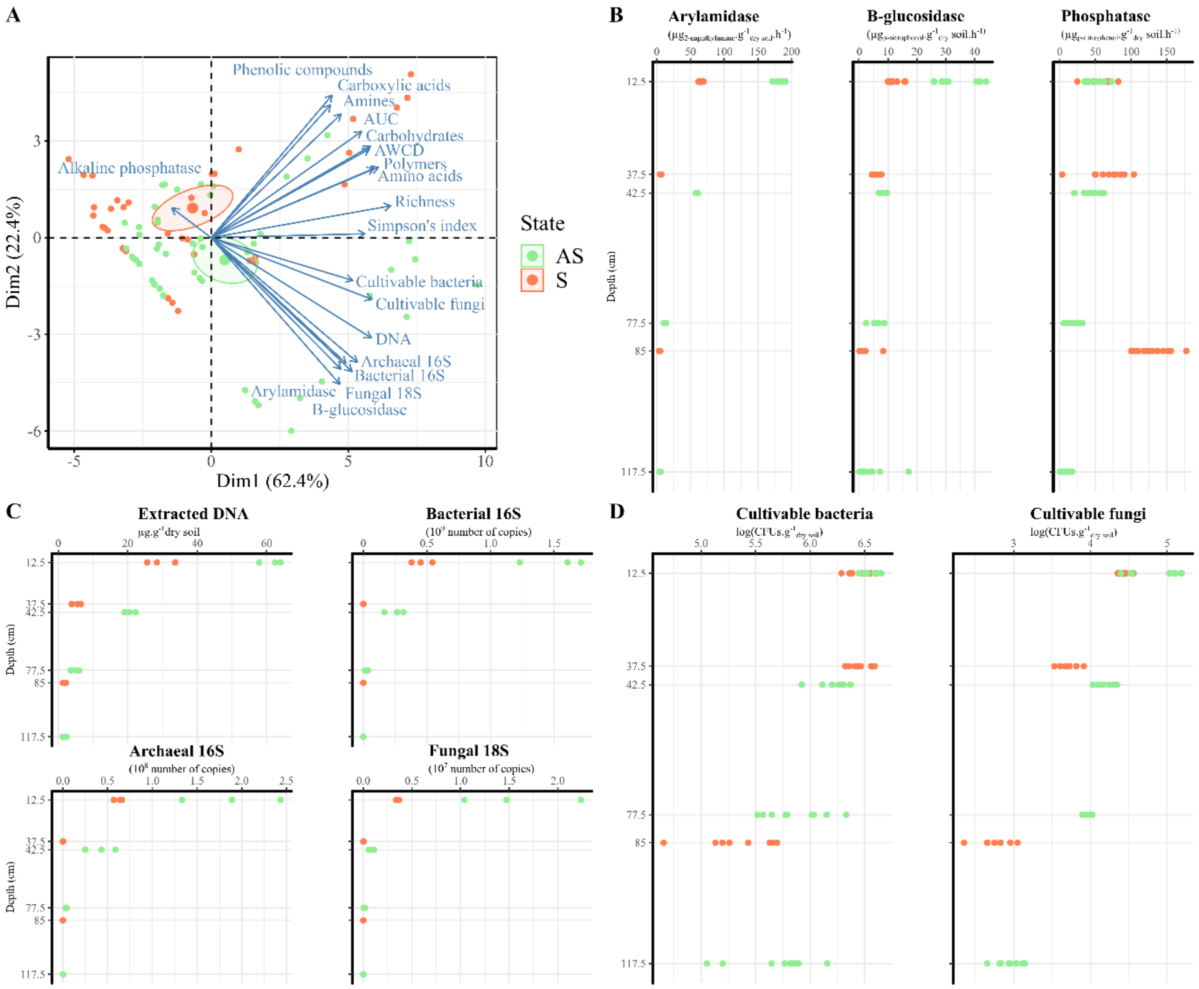

Supplementary Figure S3. Horizons profiles (0-25 = 12.5, 25-50 = 37.5, 25-60 = 42.5, 60-95 = 77.5, 50-120 = 85, and 95-140 = 117.5 cm depth) across pits made in symptomatic (orange, S) and asymptomatic (green, AS) areas. **(A)** Ordination biplot analysis of PCA for Eco-Plates measurements (n = 3) represented by Simpson's index, AWCD, AUC, functional richness, and family compounds consumed (i.e., amines, amino acids, carbohydrates, carboxylic acids, phenolic compounds, and polymers) coupled to microbial (i.e., bacteria and fungi) level of cultivable populations, enzymatic measurements (i.e., arylamidase, β-glucosidase, alkaline phosphatase), and q-PCR measurements (i.e., archaeal and bacterial 16S, and 18S rRNA genes). The size of the arrows indicates the contribution strength of the variables. Standard error ellipses show 95 % confidence areas. **(B)** Enzymatic activities (n = 5), **(C)** q-PCR measurements (n = 3), and **(D)** microbial level of cultivable populations (n = 5).

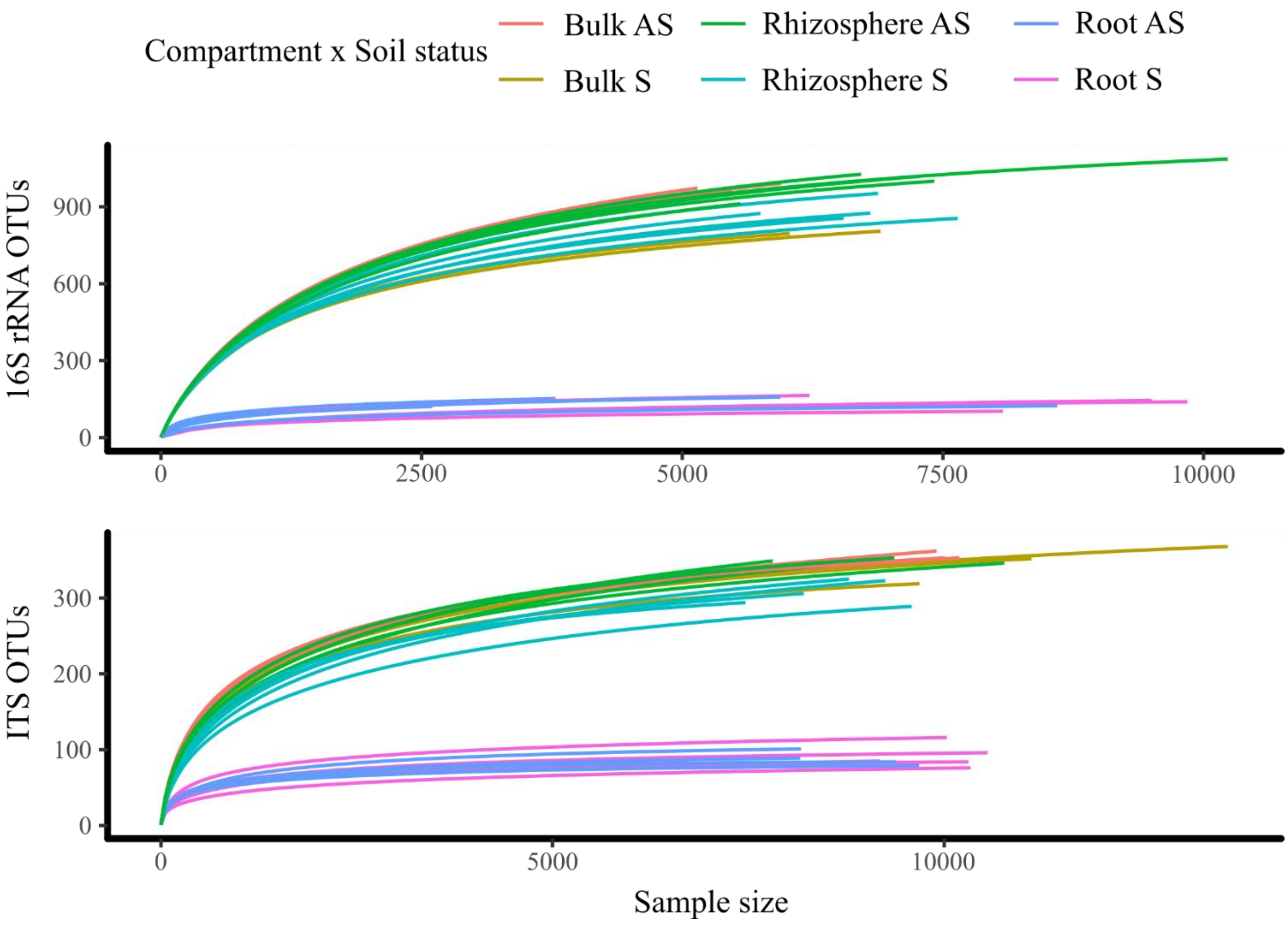

Supplementary Figure S4. Rarefaction curves of OTUs for each sample represented by Compartment × Soil status conditions for both 16S rRNA, 28S rRNA genes and ITS sequences.

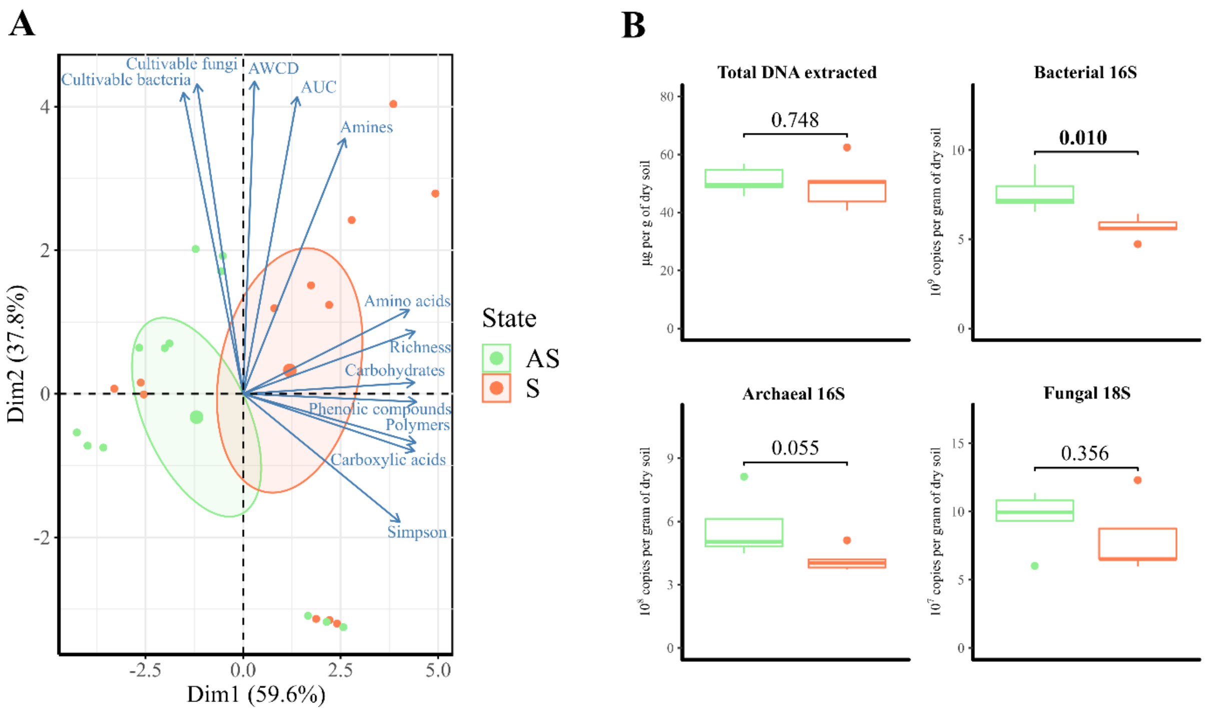

Supplementary Figure S5. Rhizosphere microbial profile in symptomatic (orange, S) and asymptomatic (green, AS) conditions. Ordination biplot analysis of PCA for **(A)** Eco-Plates measurements in rhizosphere (n = 3) represented by Simpson's index, AWCD, AUC, functional richness, and consumed family compounds coupled to bacterial and fungal level of cultivable populations (n = 5). **(B)** Total DNA extracted with associated q-PCR measurements in rhizosphere for archaeal and bacterial 16S rRNA and fungal 18S rRNA genes (n = 5). P-values, determined with t or Wilcoxon tests, depending on the normality hypothesis, are indicated.

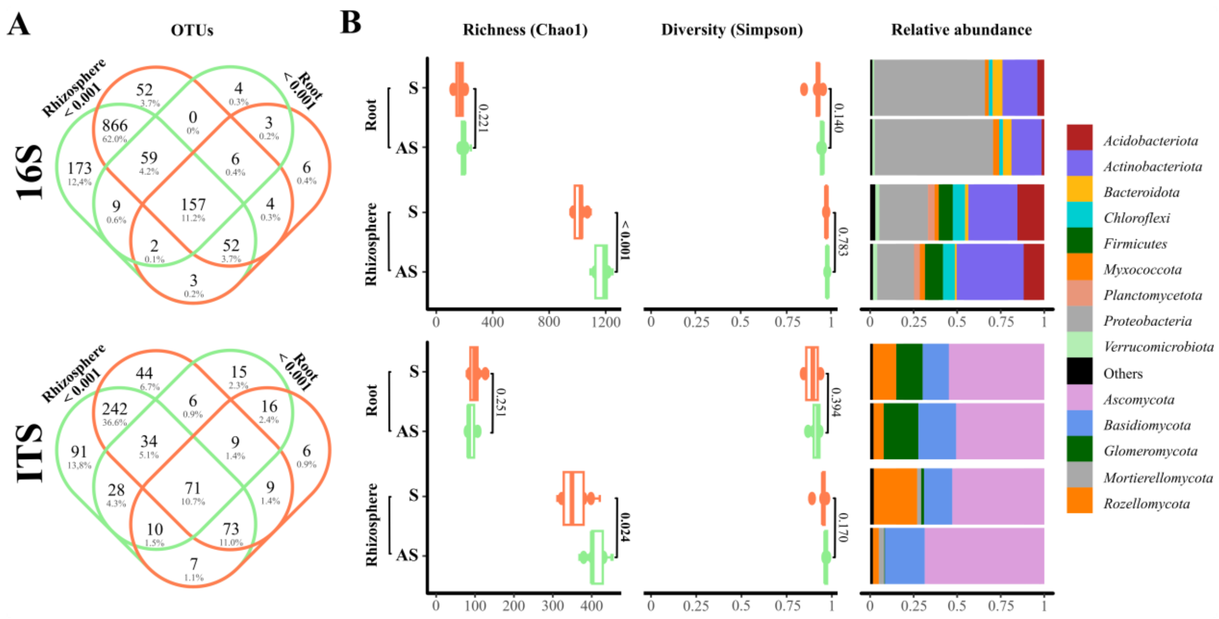

Supplementary Figure S6. Composition of the root endosphere and rhizosphere microbiome in symptomatic (orange, S) and asymptomatic (green, AS) conditions (n = 5). **(A)** Shared OTUs represented by Venn diagram for 16S and ITS sequencing, with significant overlaps detected using hyper-geometric tests. **(B)** α-diversity of both root and rhizosphere associated to their phyla. P-values, determined with t or Wilcoxon tests, depending on the normality hypothesis, are indicated.

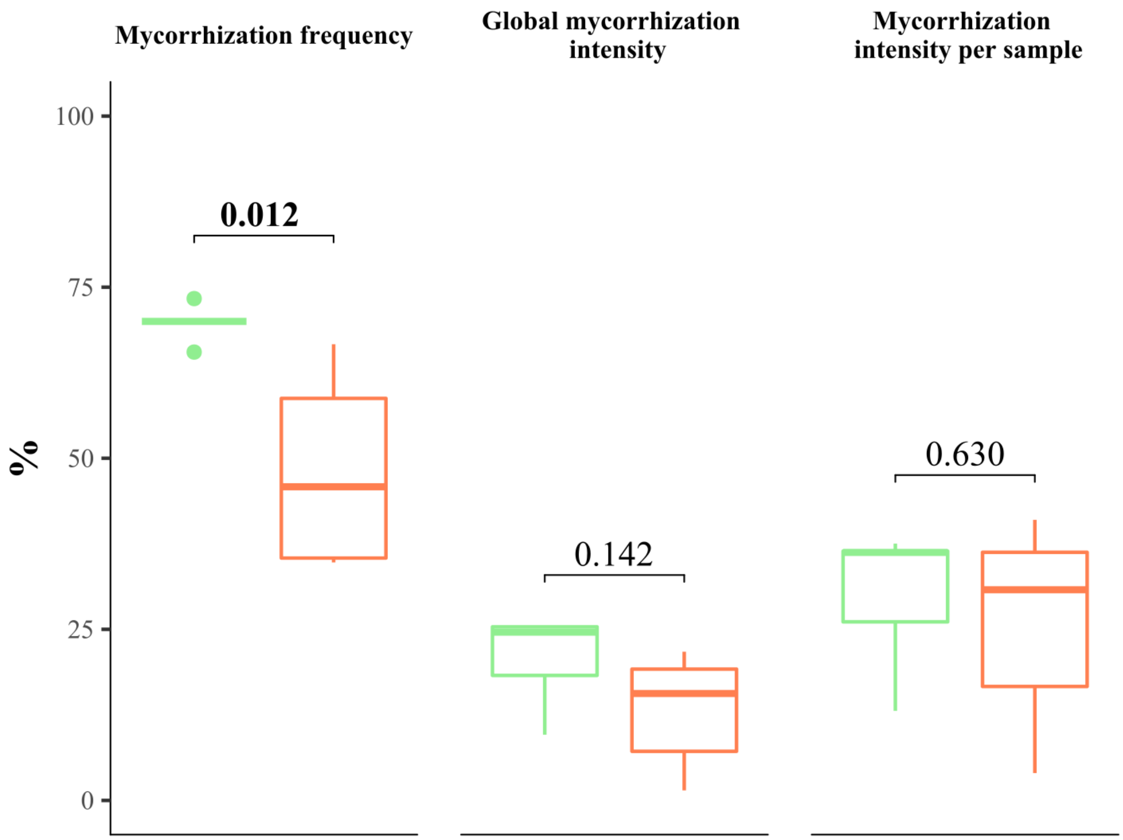

Supplementary Figure S7. Frequency and intensity of the colonization of grapevine roots by AMF (n = 5) from the studied plot with (S) and (AS) decline symptoms, as determined by black ink staining of the roots and microscopic observation. P-values, determined with t or Wilcoxon tests, depending on the normality hypothesis, are indicated.

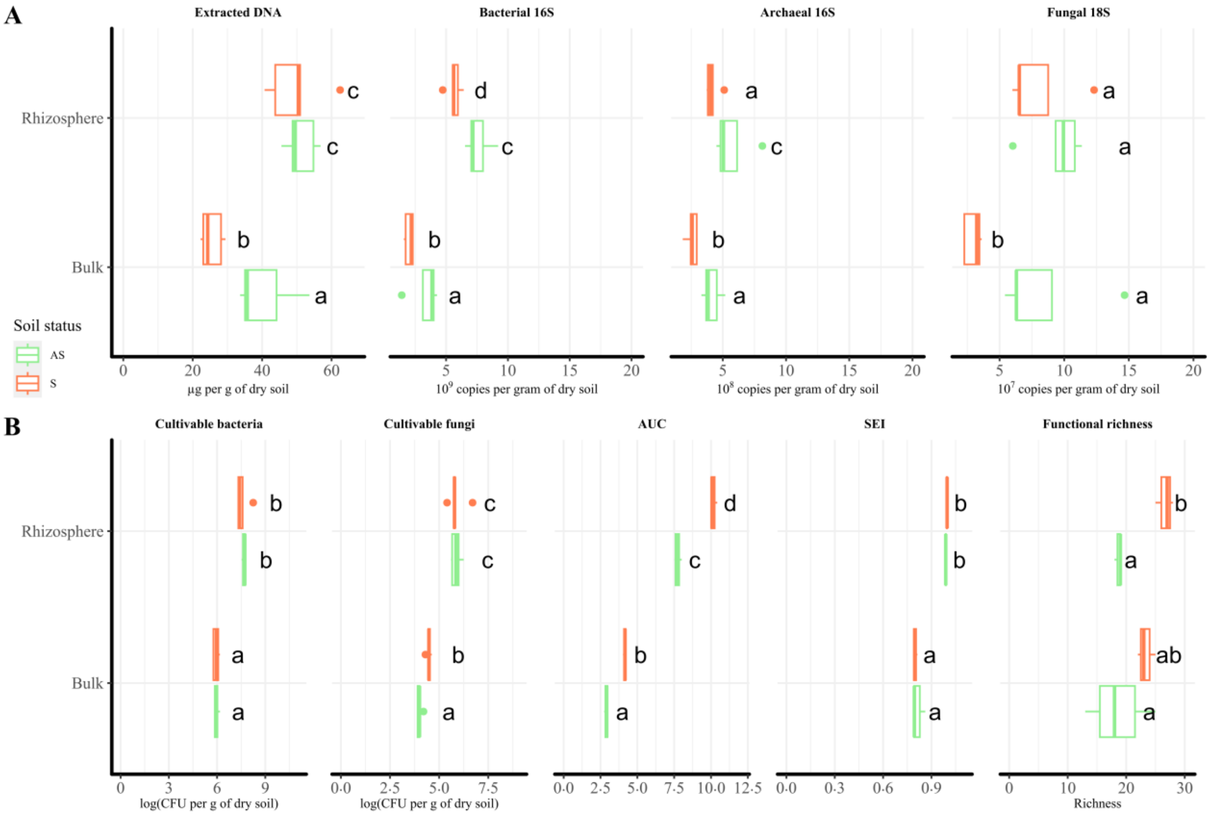

Supplementary Figure S8. Comparison of microbial profiles between the rhizosphere and bulk soils. **(A)** Comparison between DNA extracted and q-PCR measurements (i.e., number of copies of fungal 18S, bacterial and archaeal 16S genes), as well as **(B)** level of cultivable microbes and Eco-Plates measurements (SEI stands for Simpson's evenness index). Different letters indicate different groups obtained subsequently to pairwise comparisons (P < 0.05).

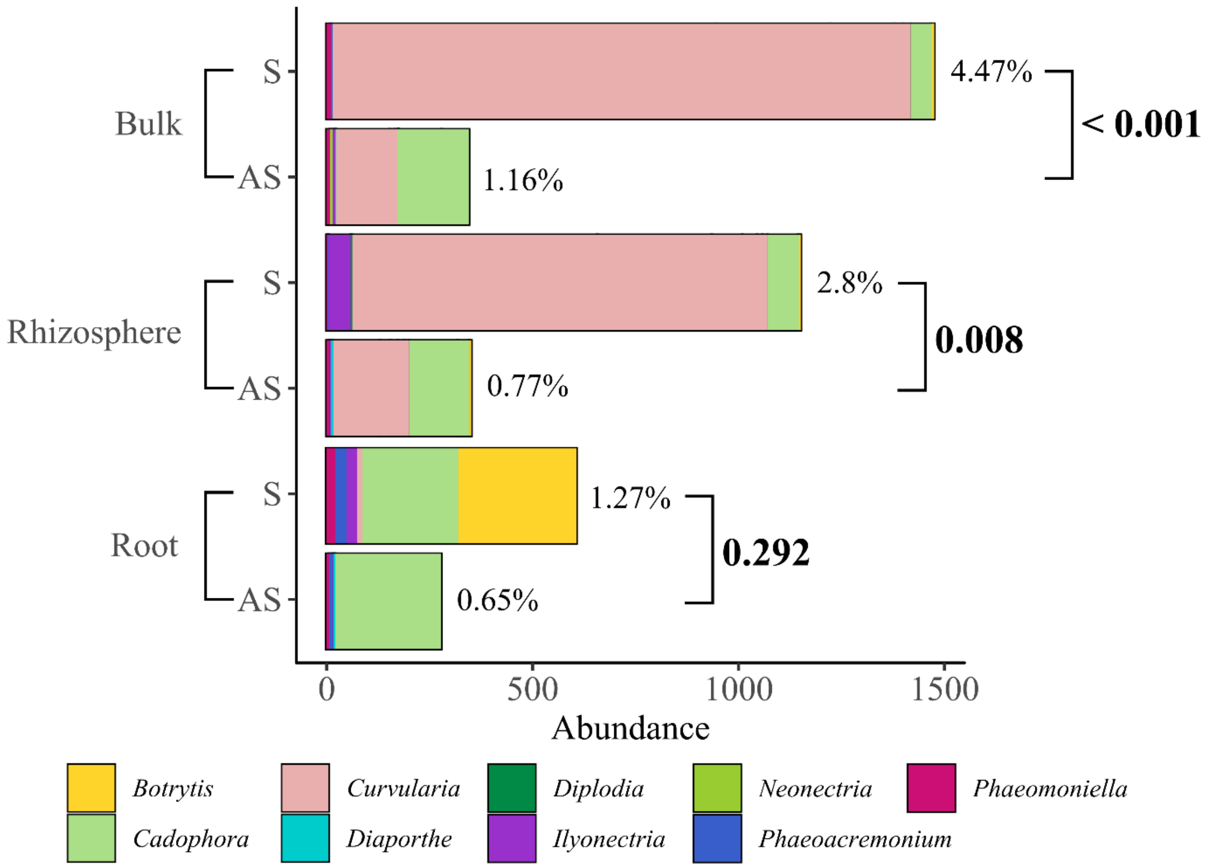

Supplementary Figure S9. Abundances of fungal OTUs potentially associated with grapevine diseases across the symptomatic and asymptomatic conditions. Percentages indicate proportions of sequences affiliated with pathogenic fungi relative to total sequences. P-values determined with t or Wilcoxon tests, depending on the normality hypothesis, are indicated.

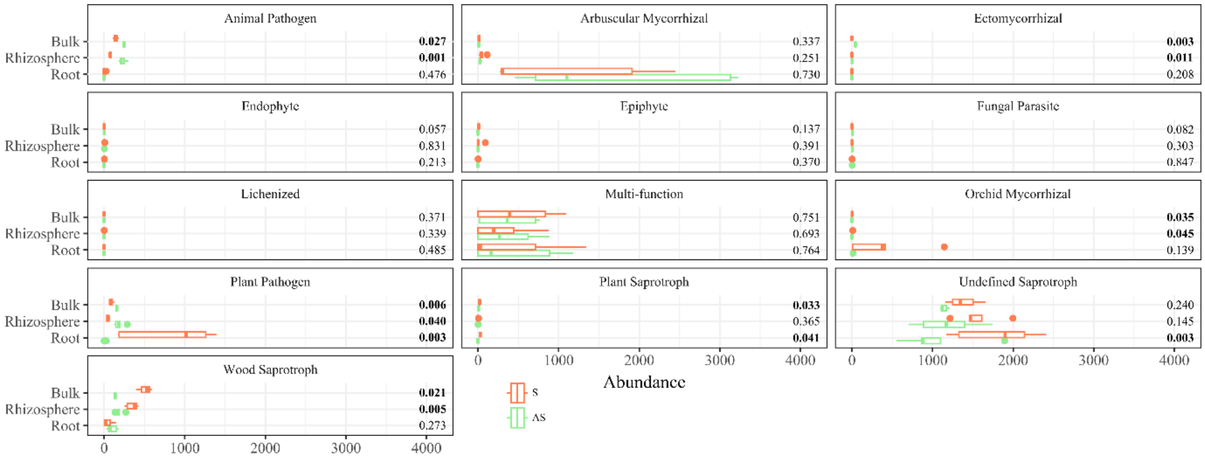

Supplementary Figure S10. Comparisons between symptomatic (S, orange) and asymptomatic (AS, green) conditions for each compartment of the 13 detected guilds from FUNGuild database. In bold are indicated significant differences based on either student t or Mann-Whitney tests.

2 Supplementary Tables

Supplementary Table S1. Primers for 16S and 18S rRNA amplification for q-PCR, as well as for 16S rRNA gene and ITS sequencing. Specific overhang Illumina adapters are in italic and underlined.

Primer	Primer sequence (5’ → 3’)	Target and size of the amplicon	Reference
**qPCR**			
**515R**	CCTACGGGAGGCAGCAG	Bacterial 16S rRNA gene (174 bp)	(López-Gutiérrez et al., 2004)
**341F**	ATTACCGCGGCTGCTGGCA		
**Arch1060R**	GGCCATGCACCWCCTCTC	Archaeal 16S rRNA gene (140 bp)	(Cadillo-Quiroz et al., 2006)
**Arch967F**	ATTGGCGGGGGAGCAC		
**FR1**	AICCATTCAATCGGTAIT	Fungal 18S rRNA gene (340 bp)	(Vainio and Hantula, 2000)
**FF390**	CGATAACGAACGAGACCT		
**Amplicons sequencing**			
**341F**	*TCGTCGGCAGCGTCAGATGTGTATAAGAGACAG*CCTACGGGNGGCWGCAG	Bacterial 16S rRNA gene V3-V4 regions (464 bp)	(Klindworth et al., 2013)
**785R**	*GTCTCGTGGGCTCGGAGATGTGTATAAGAGACAG*GACTACHVGGGTATCTAATCC		
**ITS1F**	*TCGTCGGCAGCGTCAGATGTGTATAAGAGACAG*CTTGGTCATTTAGAGGAAGTAA	Fungal ITS1 region (highly variable)	(Gardes and Bruns, 1993)
**ITS2**	*GTCTCGTGGGCTCGGAGATGTGTATAAGAGACAG*GCTGCGTTCTTCATCGATGC		(White et al., 1990)

Supplementary Table S2. Vineyard decline assessment on plot for the symptomatic (S) and asymptomatic (AS) soils. It includes yield by plant (n = 28), must composition, leaf content, and vine water status (n = 3). Numbers represent means ± standard error.

	S	AS	*P*
Yield estimation per plant			
Primary and secondary grapes number	7.8 ± 1.3	15.25 ± 2.2	**0.0160**
Primary and secondary grapes mass (g)	394 ± 110	1867 ± 378.1	**0.0009**
Bunch of grapes mass (g)	42.17 ± 6.8	104.10 ± 16.7	**0.0003**
Berry mass (g)	0.86 ± 0.03	1.40 ± 0.02	**0.0005**
Must composition			
Total soluble solids (°Brix)	23.6 ± 0.1	23.4 ± 0.1	0.44
pH	3.61 ± 0.01	3.53 ± 0.01	**0.0222**
Potential alcoholic degree	13.53 ± 0.03	13.3 ± 0.11	0.1733
Reducing sugars ($g.L^{-1}$)	228 ± 0.4	224 ± 1.6	0.1108
Total acidity ($g.L^{-1}$)	3.46 ± 0.03	3.69 ± 0.07	**0.0655**
Malic acid ($g.L^{-1}$)	1.1 ± 0.02	1.5 ± 0.06	**0.0253**
Tartaric acid ($g.L^{-1}$)	5.7 ± 0.06	6.3 ± 0.03	**0.0060**
Assimilable nitrogen ($mg.L^{-1}$)	168 ± 2.9	248 ± 1.53	**0.0001**
Alpha-amino nitrogen (NOPA) ($mg.L^{-1}$)	99 ± 3.60	120 ± 0.88	**0.0238**
Ammoniacal nitrogen ($N-NH_3$) ($mg.L^{-1}$)	32 ± 1.33	69 ± 1.20	**0.0001**
K ($mg.L^{-1}$)	1955 ± 5.24	1759 ± 18.50	**0.0056**
Petiole content ($g.kg^{-1}$ dry matter)			
K	9.24 ± 0.86	9.66 ± 1.29	0.7802
Na	0.44 ± 0.03	0.46 ± 0.04	0.7051
P	0.36 ± 0.02	0.52 ± 0.02	**0.0053**
Ca	15.25 ± 1.95	31.51 ± 0.92	**0.0058**
Mg	4.11 ± 0.57	10.4 ± 0.61	**0.0017**
N	4.96 ± 0.04	4.47 ± 0.09	**0.0208**
Leaf blade content ($g.kg^{-1}$ dry matter)			
K	8.6 ± 0.15	10.7 ± 5.23	0.7802
Na	0.26 ± 0.01	0.32 ± 0.14	0.7
P	1.28 ± 0.08	1.95 ± 0.90	0.534
Ca	22.66 ± 0.50	54.75 ± 23.02	0.1
Mg	2.56 ± 0.12	5.83 ± 2.48	0.1
N	22.61 ± 1.45	22.97 ± 3.27	0.9273
Vine water status			
$\delta^{13}C$	-26.73 ± 0.07	-25.87 ± 0.10	0.9273

Supplementary Table S3. Physicochemical characteristics of the different depth soils from the studied plot with (S) and without (AS) decline symptoms. Data shown are the values obtained after pooling 3 subsamples.

	S			AS			
Depth	0-25	25-50	50-120	0-25	25-60	60-95	95-140
Soil classification	Sand	Sand	Loamy sand	Sand	Sand	Sand	Sandy clay loam
Basic soil properties							
Color	Brown yellowish	Brown yellowish	Variegated: light gray, dark red, deep brown	Brown yellowish	Brown yellowish	Light gray	Variegated: Honest brown, gray
Root structure	No deep roots. Weed roots of all sizes and in all directions	Vertical and oblique, at the base of the horizon	Large, medium, small, all vertical and oblique	All sizes, oblique and horizontal	A few medium-sized, fine, oblique, vertical roots	A few medium-sized vertical roots	A few living roots, oblique, vertical
Compactness	Low	Low	Compact	Very compact	Compact	Slightly compact	Very compact
Clay (%)	3.7	3.7	19.2	5.7	6.8	3.3	23.8
Fine silt (%)	3.6	3.8	2.3	3.9	4.6	3.6	17.5
Coarse silt (%)	3	3.1	2.1	3.8	3.1	3.1	7.3
Fine sand (%)	12.1	21.5	9.6	13	11	11.2	6.9
Coarse sand (%)	77.6	67.9	66.8	73.6	74.5	78.8	44.5
pH (H_2O)	5.82	6.31	5.58	5.44	5.16	5.56	6.49
pH (KCl)	4.84	5.01	4.58	4.33	4.05	4.51	5.28
CEC ($cmol^+.kg^{-1}$)	1.01	0.96	4.14	2.38	2.30	0.87	4.78
C ($g.kg^{-1}$)	1.57	0.45	0.79	4.85	1.75	0.46	1.33
N ($g.kg^{-1}$)	0.27	0.22	0.24	0.53	0.31	0.19	0.37
C / N	5.81	2.07	3.32	9.09	5.64	2.42	3.63
Micro/macronutrients							
P ($g.kg^{-1}$)	0.005	<0.002	<0.002	0.018	0.005	0.002	0.003
Organic matter ($g.kg^{-1}$)	2.71	0.77	1.37	8.39	3.02	0.8	2.3
Ca ($g.kg^{-1}$)	0.10	0.10	0.52	0.17	0.20	0.06	0.84
Mg ($g.kg^{-1}$)	0.007	0.011	0.08	0.02	0.016	0.005	0.052
K ($g.kg^{-1}$)	0.02	0.01	0.03	0.06	0.02	0.01	0.04
Na ($g.kg^{-1}$)	0.005	0.003	0.016	0.014	0.003	0.002	0.02
NO_{3-} ($mg.kg^{-1}$)	0.3	<0.15	<0.15	1.51	0.26	<0.15	<0.15
NH_3-N ($mg.kg^{-1}$)	1.14	0.67	1.12	1.67	2.17	0.74	0.81
Trace elements							
Cu ($mg.kg^{-1}$)	4.66	0.58	0.5	6.16	5.03	1.30	<0.5
Fe ($mg.kg^{-1}$)	61.91	20.92	25.05	109.4	87.24	13.52	29.22
Mn ($mg.kg^{-1}$)	21.18	6.16	<0.5	13.65	50.86	6.74	2.68
Zn ($mg.kg^{-1}$)	0.60	0.53	0.23	1.61	0.95	0.50	0.35

Supplementary Table S4. Physicochemical characteristics of the inter-row soils from the studied plot with (S) and without (AS) decline symptoms. Numbers represents means ± SE (n = 3). In bold are indicated significant differences based on either student t or Mann-Whitney tests.

	S	AS	*P*
Basic soil properties			
Clay (%)	5.13 ± 0.18	5.73 ± 0.57	0.405
Silt (%)	6.3 ± 0.23	7 ± 0.25	0.110
Sand (%)	88.57 ± 0.24	87.27 ± 0.50	0.104
pH (H_2O)	6.49 ± 0.11	6.34 ± 0.03	0.376
pH (KCl)	5.55 ± 0.03	5.48 ± 0.03	0.196
IPC	0.35 ± 0.01	0.31 ± 0.01	0.066
CEC ($cmol^+.kg^{-1}$)	1.72 ± 0.11	2.57 ± 0.07	**0.001**
C ($g.kg^{-1}$)	5.7 ± 0.01	5.46 ± 0.02	0.072
N ($g.kg^{-1}$)	0.4 ± 0.01	0.45 ± 0.02	**0.001**
C / N	14.12 ± 0.12	12.22 ± 0.11	**0.001**
Micro/macronutrients			
P ($g.kg^{-1}$)	0.037 ± 0.001	0.045 ± 0.001	0.072
Organic matter ($g.kg^{-1}$)	9.86 ± 0.01	9.45 ± 0.02	0.072
Ca ($g.kg^{-1}$)	0.40 ± 0.01	0.63 ± 0.05	**0.035**
Mg ($g.kg^{-1}$)	0.02 ± 0.01	0.06 ± 0.01	0.1
K ($g.kg^{-1}$)	0.04 ± 0.01	0.06 ± 0.01	0.1
Na ($g.kg^{-1}$)	0.030 ± 0.001	0.028 ± 0.001	0.344
NO_{3-} ($mg.kg^{-1}$)	3.15 ± 0.23	5.15 ± 0.20	**0.003**
NH_3-N ($mg.kg^{-1}$)	2.81 ± 0.01	2.51 ± 0.16	0.203
Trace elements			
Cu ($mg.kg^{-1}$)	7.64 ± 0.49	9.21 ± 0.11	0.079
Fe ($mg.kg^{-1}$)	169 ± 2.65	179 ± 0.58	0.056
Mn ($mg.kg^{-1}$)	5.83 ± 0.34	15.13 ± 0.07	**0.001**
Zn ($mg.kg^{-1}$)	2.17 ± 0.14	2.42 ± 0.07	0.207

Supplementary Table S5. Cultivable population levels of bacteria and fungi, and Eco-Plates measurements (AUC, Simpson's index, family compounds consumed, and functional richness at 96 hours post-incubation) within the symptomatic (S) and asymptomatic (AS) rhizosphere and bulk soils. Means ± SE are presented for bacterial and fungal counts (n = 5), as well as for Eco-Plates measurements (n = 3). Letter [a] represents variables in log (CFUs / g of dry soil), while [b] represents variables calculated based on AWCD values from the Eco-Plates.

		S	AS	*p*
Bulk	Cultivable bacteria [a]	6.85 ± 0.04	7.10 ± 0.02	**< 0.001**
	Cultivable fungi [a]	5.47 ± 0.03	5.08 ± 0.09	**0.006**
	AUC [b]	4.14 ± 0.06	2.89 ± 0.08	**0.001**
	Simpson's index [b]	0.815 ± 0.023	0.798 ± 0.008	0.534
	Functional richness [b]	23.33 ± 0.88	18.67 ± 3.48	0.311
	Amines [b]	0.74 ± 0.74	0.40 ± 0.21	0.700
	Amino acids [b]	3.55 ± 1.45	2.76 ± 0.79	0.400
	Carbohydrates [b]	8.74 ± 1.77	3.88 ± 0.71	0.095
	Carboxylic acids [b]	4.21 ± 0.54	2.89 ± 0.83	0.263
	Phenolic compounds [b]	0.008 ± 0.01	0.52 ± 0.31	0.354
	Polymers [b]	3.54 ± 0.17	1.58 ± 0.17	0.119
Rhizosphere	Cultivable bacteria [a]	7.57 ± 0.18	7.69 ± 0.05	0.553
	Cultivable fungi [a]	5.89 ± 0.22	5.87 ± 0.12	0.952
	AUC [b]	10.16 ± 0.13	7.73 ± 0.14	**< 0.001**
	Simpson's index [b]	0.996 ± 0.002	0.989 ± 0.001	**0.046**
	Functional richness [b]	26.67 ± 0.88	18.67 ± 0.33	**0.006**
	Amines [b]	3.36 ± 0.28	2.63 ± 0.01	0.1
	Amino acids [b]	11.81 ± 1.09	7.44 ± 0.17	0.054
	Carbohydrates [b]	18.52 ± 0.11	15.75 ± 0.2	**0.001**
	Carboxylic acids [b]	12.21 ± 0.14	9.36 ± 0.23	**0.001**
	Phenolic compounds [b]	2.87 ± 0.18	1.73 ± 0.13	**0.009**
	Polymers [b]	7.77 ± 0.37	5.14 ± 0.17	**0.009**

Supplementary Table S6. Factors effects related to compartment (bulk, rhizosphere, root endosphere) and soil composition (S, AS) on richness, diversity, and β-diversity related to bacterial, fungal, and Glomeromycota communities. Significances were assessed through a Type II ANOVA for richness and diversity, while PERMANOVA (n=999) was used for distance dissimilarities. Significant P-values (<0.05) were represented in bold.

		Richness (Chao1)		Diversity (Simpson)		β-diversity (Bray-Curtis)		
		F	*P*	F	*P*	R^2	F	*P*
16S	Compartment (C)	2472.77	**<0.001**	17.17	**<0.001**	0.55	22.91	**0.001**
	State (S)	98.96	**<0.001**	3.53	0.075	0.09	7.85	**0.001**
	C × S	23.74	**<0.001**	2.23	0.133	0.11	4.66	**0.004**
ITS	C	484.47	**<0.001**	23.82	**<0.001**	0.45	14.47	**0.001**
	S	7.46	**0.013**	2.87	0.106	0.11	7.06	**0.001**
	C × S	5.03	**0.017**	0.13	0.881	0.13	4.16	**0.001**

Supplementary Table S7. Reports of α-diversities metrics represented by Chao1 and Simpson for each of the conditions among the bacterial and fungal communities. Different letters indicate significant differences among the communities (pairwise test, $p < 0.05$).

			Richness (Chao1)	Diversity (Simpson)
16S	Bulk	AS	1178 ± 10 a	0.99 ± 0.01 a
		S	931 ± 9 b	0.99 ± 0.01 ab
	Rhizosphere	AS	1174 ± 20 a	0.99 ± 0.01 a
		S	1022 ± 18 ab	0.98 ± 0.01 b
	Root	AS	186 ± 7 c	0.96 ± 0.01 b
		S	160 ± 15 c	0.93 ± 0.01 b
ITS	Bulk	AS	416 ± 15 a	0.98 ± 0.01 a
		S	378 ± 17 b	0.97 ± 0.01 a
	Rhizosphere	AS	408 ± 10 a	0.97 ± 0.01 a
		S	355 ± 15 b	0.94 ± 0.01 ab
	Root	AS	91 ± 5 c	0.91 ± 0.01 bc
		S	102 ± 7 c	0.89 ± 0.01 c

Supplementary Table S8. Abundances (± SE) in percentage of fungal OTUs potentially associated with grapevine diseases across compartment × soil status (S: Symptomatic; AS: Asymptomatic) conditions. Percentages indicate proportions of sequences affiliated with pathogenic fungi relative to total sequences. Significant differences were detected using student t-tests or Wilcoxon-tests, depending on the normality and variance (n=5). P values below 0.05 are highlighted in bold.

	Bulk			Rhizosphere			Root		
	S	AS	*P*	S	AS	*P*	S	AS	*P*
Botrytis	0.006 ± 0.012	0 ± 0	0.423	0.002 ± 0.005	0.009 ± 0.019	0.368	0.521 ± 0.162	0 ± 0	< **0.001**
Cadophora	0.167 ± 0.031	0.516 ± 0.108	**0.016**	0.146 ± 0.219	0.266 ± 0.095	0.237	0.424 ± 0.619	0.468 ± 0.397	0.88
Curvularia	4.271 ± 1.104	0.464 ± 0.075	**0.019**	1.836 ± 1.034	0.339 ± 0.147	**0.018**	0.024 ± 0.051	0 ± 0	0.290
Diaporthe	0 ± 0	0.003 ± 0.006	0.422	0 ± 0	0.007 ± 0.014	0.242	0 ± 0	0.009 ± 0.013	0.142
Diplodia	0 ± 0	0 ± 0	NA	0.007 ± 0.019	0 ± 0	0.373	0 ± 0	0 ± 0	NA
Ilyonectria	0 ± 0	0.018 ± 0.021	0.225	0.098 ± 0.104	0.005 ± 0.014	**0.017**	0.045 ± 0.070	0.003 ± 0.010	0.192
Neonectria	0 ± 0	0.021 ± 0.024	0.222	0 ± 0	0 ± 0	NA	0 ± 0	0 ± 0	NA
Phaeoacremonium	0.003 ± 0.006	0 ± 0	0.422	0.004 ± 0.006	0.002 ± 0.005	0.545	0.051 ± 0.078	0.015 ± 0.025	0.293
Phaeomoniella	0.030 ± 0.034	0.024 ± 0.024	0.786	0.004 ± 0.010	0.011 ± 0.014	0.294	0.040 ± 0.043	0.0127 ± 0.034	0.227
Total	4.467 ± 0.507	1.158 ± 0.125	< **0.001**	2.800 ± 1.287	0.775 ± 0.157	**0.008**	1.267 ± 1.134	0.655 ± 0.431	0.292

Supplementary Table S9. Variables explaining their latent variables and loadings scores used to build the first raw PLS-PM model on the left side of the table. Variables with less than 0.7 of loading scores were removed for the presented PLS-PM model, and the remaining ones are listed on the right side of the table.

Latent variables (Raw model)	Variables (Raw model)	Loadings (Raw model)	Latent variables kept (Figure 6)	Variables kept (Figure 6)
Soil status	Soil_status	1	Soil status	SoilStatus
Bulk soil physicochemistry	Clay	0.43090977	Bulk soil physicochemistry	Silt
	Silt	0.8396269		Sand
	Sand	-0.8260893		C
	C	-0.9911194		N
	N	0.97713248		Organic_matter
	Organic_matter	-0.9911194		CN
	CN	-0.9812111		pH_KCl
	pH_water	-0.6306775		P
	pH_KCl	-0.7312513		CEC
	P	0.98973515		Ca
	CEC	0.99643453		Mg
	Ca	0.92599674		K
	Mg	0.98900638		Cu
	K	0.98115812		Fe
	Na	-0.6194811		Mn
	Cu	0.91430596		Zn
	Fe	0.88752088		IPC
	Mn	0.99394085		NO
	Zn	0.7503079		NH4
	IPC	-0.8792689	Bulk soil functionality	B_Arylamidase
	NO	0.94618903		B_Bglucosidase
	NH4	-0.8050412		B_Phosphatase
Bulk soil functionality	B_Arylamidase	-0.9503709		B_AWCD
	B_Bglucosidase	-0.9357832		B_AUC
	B_Phosphatase	-0.9828306		B_Richness
	B_AWCD	0.95329211		B_Carbohydrates
	B_AUC	0.9724566		B_Phenolic_compounds
	B_Simpson_ecoplate	-0.3303272		B_Polymers
	B_Richness_ecoplate	0.73503375		B_DNA
	B_Amines	0.47547028		B_Animal Pathogen
	B_Amino_acids	0.20101765		B_Ectomycorrhizal
	B_Carbohydrates	0.91189053		B_Endophyte
	B_Carboxylic_acids	0.66835425		B_Epiphyte
	B_Phenolic_compounds	-0.7691829		B_Fungal_parasite
	B_Polymers	0.7690777		B_Multi_function
	B_Amino_acid_metabolism	0.07899317		B_Orchid_mycorrhizal
	B_Biosynthesis_of_other_secondary_metabolites	0.40791351		B_Plant_pathogen
	B_Carbohydrate_metabolism	0.50539312		B_Plant_saprotroph
	B_Energy_metabolism	0.37022424		B_Wood_saprotroph
	B_Glycan_biosynthesis_and_metabolism	0.29850711	Bulk soil bacterial communities	B_NMDS1_Bacteria
	B_Lipid_metabolism	0.07908781		B_NMDS2_Bacteria
	B_Membrane_transport	0.52941244		B_16Sa_qpcr
	B_Metabolism_of_cofactors_and_vitamins	0.26991484		B_16Sb_qpcr
	B_Metabolism_of_other_amino_acids	-0.4792434		B_Observed_16S
	B_Metabolism_of_terpenoids_and_polyketides	0.54776964	Bulk soil fungal communities	B_Cultivable_fungi
	B_Nucleotide_metabolism	0.38831728		B_ITS_qpcr

	B_Signal_transduction	0.44178427
	B_Signaling_molecules_and_interaction	0.02434216
	B_Xenobiotics_biodegradation_and_metabolism	-0.5017398
	B_DNA	-0.8188878
	B_Animal_pathogen	-0.8962569
	B_Arbuscular_mycorrhizal	0.66319053
	B_Ectomycorrhizal	-0.974755
	B_Endophyte	-0.9541954
	B_Epiphyte	0.77247093
	B_Fungal_parasite	-0.8788545
	B_Multi_function	0.73820473
	B_Orchid_mycorrhizal	0.9066457
	B_Plant_pathogen	-0.9775023
	B_Plant_saprotroph	0.95265634
	B_Undefined_saprotroph	0.68323158
	B_Wood_saprotroph	0.9843225
Bulk soil bacterial communities	B_NMDS1_bacteria	-0.9809264
	B_NMDS2_bacteria	-0.9823998
	B_Cultivable_bacteria	-0.0272007
	B_16Sa_qpcr	0.8705481
	B_16Sb_qpcr	0.75546874
	B_Observed_16S	0.97506706
	B_Simpson_16S	0.25148588
Bulk soil fungal communities	B_Cultivable_fungi	-0.9599745
	B_ITS_qpcr	0.80909495
	B_Observed_ITS	0.43273744
	B_Simpson_ITS	0.9807446
	B_NMDS1_fungi	-0.9474074
	B_NMDS2_fungi	0.99350794
Rhizosphere functionality	RH_Animal_pathogen	-0.8622227
	RH_Arbuscular_mycorrhizal	0.52940906
	RH_Ectomycorrhizal	-0.8033268
	RH_Endophyte	0.58279348
	RH_Epiphyte	0.38253101
	RH_Fungal_parasite	-0.2916087
	RH_Multi_function	-0.2998235
	RH_Orchid_mycorrhizal	0.64876659
	RH_Plant_pathogen	-0.8214435
	RH_Undefined_saprotroph	0.71325158
	RH_Wood_saprotroph	0.84047044
	RH_Amino_acid_metabolism	-0.7950351
	RH_Biosynthesis_of_other_secondary_metabolites	-0.7820878
	RH_Carbohydrate_metabolism	-0.8616925
	RH_Energy_metabolism	-0.2640451
	RH_Glycan_biosynthesis_and_metabolism	-0.2578567
	RH_Lipid_metabolism	0.46834114
	RH_Membrane_transport	-0.1081123

	B_Simpson_ITS
	B_NMDS1_fungi
	B_NMDS2_fungi
Rhizosphere functionality	RH_Animal_pathogen
	RH_Ectomycorrhizal
	RH_Plant_pathogen
	RH_Undefined_saprotroph
	RH_Wood_saprotroph
	Rh_Amino_acid_metabolism
	Rh_Biosynthesis_of_other_secondary_metabolites
	Rh_Carbohydrate_metabolism
	RH_AWCD
	RH_AUC
	RH_Simpson_ecoplate
	RH_Richness_ecoplate
	RH_Amines
	RH_Amino_acids
	RH_Carbohydrates
	RH_Carboxylic_acids
	RH_Phenolic_compounds
	RH_Polymers
Rhizosphere bacterial communities	RH_16Sb_qpcr
	RH_Observed_16S
	RH_NMDS1_bacteria
Rhizosphere fungal communities	RH_Observed_ITS
	RH_NMDS1_Fun
	RH_NMDS2_Fun
Root functionality	Rt_Amino_acid_metabolism
	Rt_Carbohydrate_metabolism
	Rt_Glycan_biosynthesis_and_metabolism
	Rt_Lipid_metabolism
	Rt_Metabolism_of_cofactors_and_vitamins
	Rt_Metabolism_of_other_amino_acids
	Rt_Nucleotide_metabolism
	Rt_Xenobiotics_biodegradation_and_metabolism
	Rt_Simpson_16S
Root bacterial communities	Rt_NMDS1_bacteria
	Rt_NMDS2_bacteria
Root fungal communities	Rt_NMDS1_fungi
	Rt_NMDS2_fungi
Leaves	Petiole_P
	Petiole_Ca
	Petiole_Mg
	Petiole_N
	Limb_K
	Limb_Na
	Limb_P

	RH_Metabolism_of_cofactors_and_vitamins	-0.0599826
	RH_Metabolism_of_other_amino_acids	0.09209966
	RH_Metabolism_of_terpenoids_and_polyketides	-0.4811856
	RH_Nucleotide_metabolism	-0.4965983
	RH_Signal_transduction	-0.3155963
	RH_Signaling_molecules_and_interaction	-0.6064522
	RH_Xenobiotics_biodegradation_and_metabolism	0.57316413
	RH_DNA	-0.0099413
	RH_AWCD	0.97548214
	RH_AUC	0.98012971
	RH_Simpson_ecoplate	0.93857255
	RH_Richness_ecoplate	0.9840347
	RH_Amines	0.87859005
	RH_Amino_acids	0.93566523
	RH_Carbohydrates	0.97764522
	RH_Carboxylic_acids	0.97333258
	RH_Phenolic_compounds	0.96625138
	RH_Polymers	0.95234551
Rhizosphere bacterial communities	RH_16Sa_qpcr	0.63403285
	RH_16Sb_qpcr	0.78978077
	RH_Observed_16S	0.82709822
	RH_Simpson_16S	0.39276121
	RH_Cultivable_bacteria	0.51731347
	RH_NMDS1_bacteria	-0.9566107
	RH_NMDS2_bacteria	-0.216761
Rhizosphere fungal communities	RH_ITS_qpcr	0.23927348
	RH_Cultivable_fungi	0.09667491
	RH_Observed_ITS	0.96594722
	RH_Simpson_ITS	0.57352059
	RH_NMDS1_fungi	-0.8740281
	RH_NMDS2_fungi	0.93584416
Root functionality	Rt_Amino_acid_metabolism	0.78499766
	Rt_Biosynthesis_of_other_secondary_metabolites	0.58340543
	Rt_Carbohydrate_metabolism	0.89365342
	Rt_Energy_metabolism	0.61967343
	Rt_Glycan_biosynthesis_and_metabolism	0.81855792
	Rt_Lipid_metabolism	-0.7315463
	Rt_Membrane_transport	0.18186607
	Rt_Metabolism_of_cofactors_and_vitamins	0.91269411
	Rt_Metabolism_of_other_amino_acids	-0.7482361
	Rt_Metabolism_of_terpenoids_and_polyketides	-0.6375607
	Rt_Nucleotide_metabolism	0.98054871
	Rt_Signal_transduction	0.41116546
	Rt_Xenobiotics_biodegradation_and_metabolism	-0.9325061
	Rt_Animal_pathogen	-0.079679
	Rt_Arbuscular_mycorrhizal	0.54655161
	Rt_MultiAffiliation	-0.2715014
	Rt_Orchid_mycorrhizal	-0.5303049
	Rt_Plant_pathogen	-0.493544
	Rt_Undefined_saprotroph	0.01803256

	Limb_Ca
	Limb_Mg
Berry	Berry_mass
	Berry_Reducing_sugars
	Berry_Degree
	Berry_Total_acidity
	Berry_pH_must
	Berry_Malic_acid
	Berry_Tartric_acid
	Berry_Assimilable_N
	Berry_NOPA
	Berry_NNH3
	Berry_K_must
	Berry_DeltaC13
	Berry_Volatile_acidity

	Rt_Wood_saprotroph	0.59125785
Root bacterial communities	Rt_Observed_16S	0.39943465
	Rt_Simpson_16S	0.927922
	Rt_NMDS1_bacteria	0.92208385
	Rt_NMDS2_bacteria	-0.9333578
Root fungal communities	Rt_F	0.41538472
	Rt_M	0.6693177
	Rt_m	0.62646599
	Rt_Observed_ITS	-0.1333519
	Rt_Simpson_ITS	0.43526644
	Rt_NMDS1_fungi	0.87018053
	Rt_NMDS2_fungi	0.88828783
Leaves	Petiole_K	0.6032486
	Petiole_Na	0.04715637
	Petiole_P	0.96002308
	Petiole_Ca	0.81967355
	Petiole_Mg	0.8526825
	Petiole_N	-0.9508764
	Limb_K	0.74281829
	Limb_Na	0.76793325
	Limb_P	0.8387743
	Limb_Ca	0.94939271
	Limb_Mg	0.94104602
	Limb_N	-0.1058894
Berry	Berry_mass	0.98734762
	Berry_Brix	-0.4179997
	Berry_Reducing_sugars	-0.8281621
	Berry_Degree	-0.7395836
	Berry_Total_acidity	0.88531339
	Berry_pH_must	-0.9544135
	Berry_Malic_acid	0.95116344
	Berry_Tartric_acid	0.98278809
	Berry_Assimilable_N	0.994208
	Berry_NOPA	0.9531086
	Berry_NNH3	0.99374799
	Berry_K_must	-0.9854988
	Berry_DeltaC13	0.96713659
	Berry_Volatile_acidity	-0.9279746

3 References

Cadillo-Quiroz, H., Bräuer, S., Yashiro, E., Sun, C., Yavitt, J., and Zinder, S. (2006). Vertical profiles of methanogenesis and methanogens in two contrasting acidic peatlands in central New York State, USA. Environ. Microbiol. 8, 1428–1440. doi: 10.1111/j.1462-2920.2006.01036.x

Gardes, M., and Bruns, T. D. (1993). ITS primers with enhanced specificity for basidiomycetes - application to the identification of mycorrhizae and rusts. Mol. Ecol. 2, 113–118.

Gollotte, A., van Tuinen, D., and Atkinson, D. (2004). Diversity of arbuscular mycorrhizal fungi colonising roots of the grass species Agrostis capillaris and Lolium perenne in a field experiment. Mycorrhiza 14, 111–117. doi: 10.1007/s00572-003-0244-7

Klindworth, A., Pruesse, E., Schweer, T., Peplies, J., Quast, C., Horn, M., et al. (2013). Evaluation of general 16S ribosomal RNA gene PCR primers for classical and next-generation sequencing-based diversity studies. Nucleic Acids Res. 41, 1–11. doi: 10.1093/nar/gks808

López-Gutiérrez, J. C., Henry, S., Hallet, S., Martin-Laurent, F., Catroux, G., and Philippot, L. (2004). Quantification of a novel group of nitrate-reducing bacteria in the environment by real-time PCR. J. Microbiol. Methods 57, 399–407. doi: 10.1016/j.mimet.2004.02.009

Suzuki, K., Takahashi, K., and Harada, N. (2020). Evaluation of primer pairs for studying arbuscular mycorrhizal fungal community compositions using a MiSeq platform. Biol. Fertil. Soils 56, 853–858.

Vainio, E. J., and Hantula, J. (2000). Direct analysis of wood-inhabiting fungi using denaturing gradient gel electrophoresis of amplified ribosomal DNA. Mycol. Res. 104, 927–936. doi: 10.1017/S0953756200002471

White, T. J., Bruns, T. D., Lee, S. B., and Taylor, J. W. (1990). "Amplification and direct sequencing of fungal ribosomal RNA genes for phylogenetics," in PCR protocols: a guide to methods and applications, eds. M. A. Innis, D. H. Gelfand, J. J. Sninsky, and T. J. White (United States: Academic Press, Inc.), 315–322.
